# XHIC-Net: An Explainable Hybrid Involution–Convolution Network for Blood Smear Cell Morphology Classification

**DOI:** 10.3390/bioengineering13080938

**Published:** 2026-08-19

**Authors:** Irshad Ahmad, Muhammad Sheraz Khan, Omar Alruwaili

**Affiliations:** 1 Department of Computer Science, Islamia College Peshawar, Peshawar 25000, Khyber Pakhtunkhwa, Pakistan; msherazkhan8888527@gmail.com; 2 Department of Computer Engineering and Networks, College of Computer and Information Science, Jouf University, Sakakah 72622, Al-Jouf, Saudi Arabia

**Keywords:** leukocytes, Involution Neural Network, deep learning, medical image classification, residual block, explainable AI

## Abstract

Accurate morphological analysis of blood smears is vital for hematological diagnosis, yet manual examination is labor-intensive and subjective. While deep learning offers automation, its black-box nature and computational demands often hinder clinical trust and deployment. We propose XHIC-Net, an Explainable Hybrid Involution–Convolution Network designed for efficient and transparent cell classification. By integrating spatially adaptive involution operations with convolutional layers within a residual framework, XHIC-Net captures both contextual and fine-grained features efficiently. To enhance interpretability, a Grad-CAM-based explainable AI (XAI) module visualizes the cellular regions driving model predictions. The proposed framework was evaluated on a dataset comprising 12,879 microscopic blood smear images belonging to 12 morphological cell categories. Experimental results demonstrate that XHIC-Net achieves an overall accuracy of 98.88%, precision of 98.89%, recall of 98.87%, F1-score of 0.9887, and Cohen’s Kappa score of 0.9887. It outperformed established models, including DL models such as EfficientNetV2S, MobileNet family, DenseNet family, and VGG16, while using fewer parameters and requiring shorter training times. Furthermore, the XAI maps consistently highlighted biologically relevant structures, validating the model’s decision-making process. XHIC-Net is a strong, effective, and clear research model for automated hematology. With future clinical validation, it has the potential to be modified for point-of-care diagnostics in healthcare settings with limited resources.

## 1. Introduction

Healthcare is an abundant data space, and medical images are part of it. As imaging capabilities and computational resources have improved, the demand for sophisticated image interpretation equipment has increased. Improvements in image interpretation lead to greater diagnostic accuracy and lower healthcare costs, yielding better patient outcomes [1]. White blood cells are important in fighting diseases and maintaining health. Different subclasses of white blood cells perform specialized functions in the immune response [2]. Blood cancer, or leukemia [3], originates in the bone marrow and is characterized by an abnormally high or atypical concentration of leucocytes (white blood cells) in the bloodstream [4].

According to the American Cancer Society’s (ACS) 2019 fact sheets, a total of 176,200 new cases of leukemia, lymphoma, and myeloma were reported, with leukemia accounting for 35% (61,780 cases). Acute lymphoblastic leukemia (ALL) occurs when blasts, or immature blood cells, proliferate in the bone marrow. Unlike malignant cells from solid organs or peripheral blood, these abnormal cells originate in the bone marrow [5]. The decreased leukocyte count is due to either cessation of production in the bone marrow or destruction by infection. Leukocytosis is an increase in the number of leukocytes beyond the normal range associated with respiratory disease and indicates the presence of infection or inflammation [6]. The categorization of leukocytes depends on identifying the particular cell type affected and its associated functional or morphological abnormalities [7]. The exact subtype of abnormal white blood cells must be correctly identified, regardless of whether the total white blood cell count is elevated or suppressed [2].

Recent computational advances have supported improved hematological analysis and related diagnostic pipelines. These include multiview graph learning frameworks for drug interaction prediction relevant to leukemia treatment [8] and systematic evaluations of genome information processing techniques with potential applications in blood disorder diagnostics [9]. Such works highlight the growing role of advanced machine learning and genomic tools in complementing morphological analysis of blood smears.

Recent advancements in machine learning (ML) have led to the development of automatic leukocyte identification algorithms, particularly artificial neural networks (ANNs). These approaches are particularly relevant to leukocyte classification in general [10,11]. LeuFeatx uses a two-step approach and a pre-trained VGG16 model to classify leukocytes in leukemia diagnosis [12]. This approach achieves 96.15% accuracy on the ALL_IDB2 database. However, some studies have found lower diagnostic accuracy. Recently, research in this field has focused on DL networks and artificial intelligence. These networks have large quantities of training data. Additionally, the deep networks used in prior studies often require complex, multistage architectures and are computationally expensive, thus necessitating costly hardware [13]. The incorporation of expert domain knowledge enables nuanced morphological interpretations that are in accordance with WHO guidelines and help validate AI results in more complex scenarios, including rare subtypes.

Precise and rapid classification of white blood cells (WBCs) is critical for proper disease diagnosis and treatment. Convolutional neural network (CNN) approaches have been widely adopted to address this issue, as they enable the early detection of abnormalities, including blasts, and facilitate the monitoring of targeted therapies and treatment responses. Although CNNs are highly accurate, substantial improvements depend on generating deep features at various architectural levels. Feature selection methods lead to more discriminative features in lower-dimensional spaces, thereby improving classification accuracy. Nevertheless, due to the spatially agnostic nature of CNNs [14], channel-level relevance representations are neglected in CNNs, and inter-channel correlations fail to be captured. Recent developments have moved beyond conventional CNN architectures towards more efficient and interpretable architectures for blood cell classification. To this end, Karadd et al. [15] introduced a lightweight custom deep neural network (CDNN) comprising residual blocks and depth concatenation. This achieved an accuracy of 99.64% on the BCCD dataset using only 1.31 million parameters, demonstrating that compact networks can be as effective as deep networks for white blood cell (WBC) classification. The accuracy of DenseNet-201, EfficientNet, and ConvNeXt can be boosted further by using ensemble strategies to reach 97.8% on various WBC datasets. Visualizations generated by Grad-CAM can also increase clinician confidence [15]. In parallel, generative methods such as diffusion models have been developed for blood cell morphology classification. CytoDiffusion has been shown to excel in terms of anomaly detection, domain shift robustness, and low-data regimes, as well as generating synthetic images that are almost indistinguishable from real ones [16]. Self-attention models, such as Vision Transformer models, have also been shown to perform well on blood cell datasets [17]. These advances highlight the field’s shift towards lightweight, explainable, and clinically deployable models, which motivates our hybrid involution–convolution design. We propose a technique that uses a CNN framework optimized to improve image classification via a hybrid convolution–involution approach. In this paper, we present the following contributions:

- The XHIC-Net, an Explainable Hybrid Involution–Convolution Network, is proposed, which combines the spatial-resolution advantages of the involution with well-established feature-extraction properties. With 98.88% accuracy on a 12-class dataset comprising 12,879 micrographs, XHIC-Net surpasses EfficientNetV2S (98.4%) and MobileNetV3Large (98.4%), while using fewer parameters and reducing computational burden during capture.
- A coupled large-scale dataset is constructed from several publicly available datasets, refined to ensure class balance and image quality, and creates a strong basis for multiclass cell counting of leukocyte and non-leukocyte blood smear cells.
- In addition, we suggest possible enhancements, such as variable kernel sizes and longer normalization. Such modifications give you a 2–3% improvement in correctness and speed up convergence by 15%, which allows for further optimization.
- Albeit each is governed by the intrinsic limitations of manual analysis and conventional DL, this strategy establishes a solid foundation for accurate and effective hematology diagnosis, evidencing a potential boost to progress in wide medical image classification.

## 2. Related Work

It is crucial to distinguish between different types of leukocytes in order to diagnose blood diseases such as leukemia. This process has been extensively studied using both traditional and computational methods. This section reviews relevant work related to classical methods, CNN, and transfer learning. It summarizes their merits and demerits as a baseline for our proposed model, XHIC-Net.

Leukocytes were initially identified by visually inspecting blood smears under a microscope to examine cellular morphology, including color, shape and texture. These manual techniques are time-consuming and unreproducible because they depend on an individual’s medical know-how. To overcome these limitations, automated image analysis techniques have emerged, including segmentation and morphological feature extraction, to reduce the time spent on the process. For instance, Wang et al. [18] used a spectral and morphological framework that provided accurate results on differentiating complex intercellular heterogeneities. Dorini et al. [19] then developed a multi-scale analysis approach based on an image pyramid and applied it to white blood cell (WBC) segmentation to improve segmentation performance. Nonetheless, the advantage of this method relies on manually designed features, which are inadequate for classification or embedding generation. These classical approaches are not very robust. They require a lot of manual preprocessing, which limits their applicability to different datasets. Consequently, conventional white blood cell examination is still labor-intensive and time-consuming [20]. Yingying Ding et al. [21] proposed a two-stage classification method based on shape features, using multi-model fusion with ResNet to improve leukocyte classification accuracy. Then, SVM has been exploited to classify lymphocytes with high accuracy based on texture features. Saidani O et al. [22] emphasized the considerable challenge of categorizing WBC types from microscopic images.

Recent advances in deep learning using convolutional neural networks (CNNs) have automated feature extraction, greatly improving leukocyte classification performance. Siddique et al. [23] used the Squeeze-Net CNN architecture to categorize leukocytes into four classes, achieving 93.8% accuracy on the test dataset. Ghosh et al. [24] proposed a morphological-feature-based classifier for four kinds of leukocytes, applying a self-designed CNN, two well-known deep neural networks (DenseNet121 and ResNet50), and a Learning Classifier with minimum entropy combiner (LEC) network as an integrator of results produced to achieve 96.67% accuracy. Baghel et al. [25] report that the high accuracy rate of white blood cell recognition using WBCs-Net is computationally intensive during testing. Razzak and Naz [26] utilized a deep, contour-aware CNN along with extreme machine learning. The model achieved very high segmentation accuracy, but this came at the expense of complex preprocessing. Although CNN-based methods are highly accurate, they tend to require complex computational models and are not necessarily well-suited to multiclass classification across different datasets. Di Ruberto et al. [27] used microscopic blood image datasets from the public domain and [27] achieved an accuracy of 94.1% in detecting white blood cells and classifying them as leukemic or normal. Ferhat Bozkurt et al. [28] addressed the challenge of blood cell categorization using a deep convolutional neural network, which remains a significant issue in blood diagnosis. Emrah ASLAN et al. [29] used Ridge feature selection methods to select the most significant features, along with Maximal Information Coefficient and CNNs, to accelerate classification. Channabasava Chola et al. [30] assessed the viability and reliability of BCNet through multiple tests, including 5-fold cross-validation. Dasariraju et al. [31] classified immature leukocytes into four categories, achieving 93.45% accuracy using the random forest method.

Transfer learning with pretrained models has been widely adopted to improve leukocyte classification, especially in contexts with limited data. Despite these advances, current knowledge distillation approaches that rely on transfer learning are often inadequate for small datasets with limited labeling. Vogado et al. [32] proposed a CNN combined with SVM for leukemia classification, which achieved 96.15% accuracy on the ALL IDB2 dataset. Pretrained models provide powerful high-level visual representations learned from large-scale datasets like ImageNet that can reduce overfitting and promote generalization with few annotated hematology images. Habibzadeh et al. [33] employed pretrained ResNet and Inception architectures, achieving good performance on white blood cell classification but with increased training time. Ahmad et al. [34] have used entropy-controlled deep feature optimization that reached up to 98% for some leukocyte types, but again, the small training set prevented validation of these results. Katar and Yildirim [35] developed an interpretable vision transformer model that achieved 98.5% accuracy but required significant computational resources. Ma et al. [36] improved classification performance by integrating ResNet with a deep convolutional generative adversarial network (DC-GAN) and training the CNN for 200 iterations. Andrea Loddo et al. [37] showed that convolutional neural networks outperform traditional machine learning methods when combined with manually engineered descriptors. However, simpler techniques can also achieve high accuracy with appropriate image data. Banik et al. [38] introduced a CNN model with a dropout layer to address overfitting, reporting an average accuracy of 96% on the BCCD test database. C. Zonyfar et al. [39] presented CAB-Net, an end-to-end deep learning framework that integrates a CNN with an attention mechanism and bidirectional long short-term memory (BLSTM). Mosabbir et al. [40] applied CNNs to the complex National Institutes of Health (NIH) dataset, achieving 97.92% accuracy. Sharma et al. [41] reported a CNN-based approach achieving 87% accuracy for multiclass classification and 96% for binary classification.

In recent years, Vision Transformers (ViT) [17], ConvNeXt [42], EfficientNetV2 [43], and hybrid CNN-transformer architectures [44] have been increasingly used for blood cell classification. Ayesha et al. [17] obtained 99% accuracy when training a ViT-SAM-V hybrid on 5000 images of blood cells. Huy et al. [44] proposed ModifiedNet, which integrates depthwise separable convolutions, dilated convolutions, and residual Inception blocks, achieving 97.24% accuracy for five leukemia classes. Wisaeng et al. [43] used EfficientNetV2-S with CBAM attention and Focal Loss for B-ALL classification, achieving an accuracy of 96.11%. Multi-scale Efficient Multi-Scale Attention (EMAResDrop) was presented by Yang et al. [45] with 95.4% on ALL genetic subtypes. Hasanah et al. [46] proposed a mobile, web-based prototype for the automated detection of abnormal red blood cells (RBCs) using MobileNetV2, achieving 89.77% accuracy on six RBC morphologies. Progress in this area demonstrates a trend in the field towards attention mechanisms and efficient architectures, which motivated our hybrid evolution–convolution design.

Existing approaches present notable limitations. Instead, methods that depend on handcrafted features are typically not very robust because they rely heavily on designing specific and relevant features manually. On the other hand, several CNNs and transfer learning methods needed immense computing resources and did not perform well on very large, diverse leukocyte datasets. Additionally, the vast majority of existing methods bin data into a few categories, restricting their applications for detailed diagnostics. To address this issue, a lightweight XHIC-Net, an Explainable Hybrid Involution–Convolution Network, is proposed. XHIC-Net achieves an accuracy of up to 98.88% on a 12-class blood smear cell dataset (combining both leukocytes and non-leukocytes), while requiring fewer parameters and less training time. This makes it scalable for clinical use.

## 3. Materials and Methods

In this section, we delve into the technical details of our study, covering the dataset specifications, the design of the proposed XHIC-Net architecture, and its components.

### 3.1. Dataset

In this study, two publicly available high-quality datasets are combined to form a clinically relevant 12-class dataset for multiclass classification of leukocytes and non-leukocyte blood smear cells. The first dataset, Acevedo et al. [47], consists of 17,092 images from normal peripheral blood cells acquired in the Core Laboratory at the Hospital Clinic of Barcelona (2015–2019). The second dataset is Matek et al. [48], which contains 171,374 single-cell images from bone marrow smears of 945 patients with different hematological disorders at the Munich Leukemia Laboratory.

**Figure 1:**
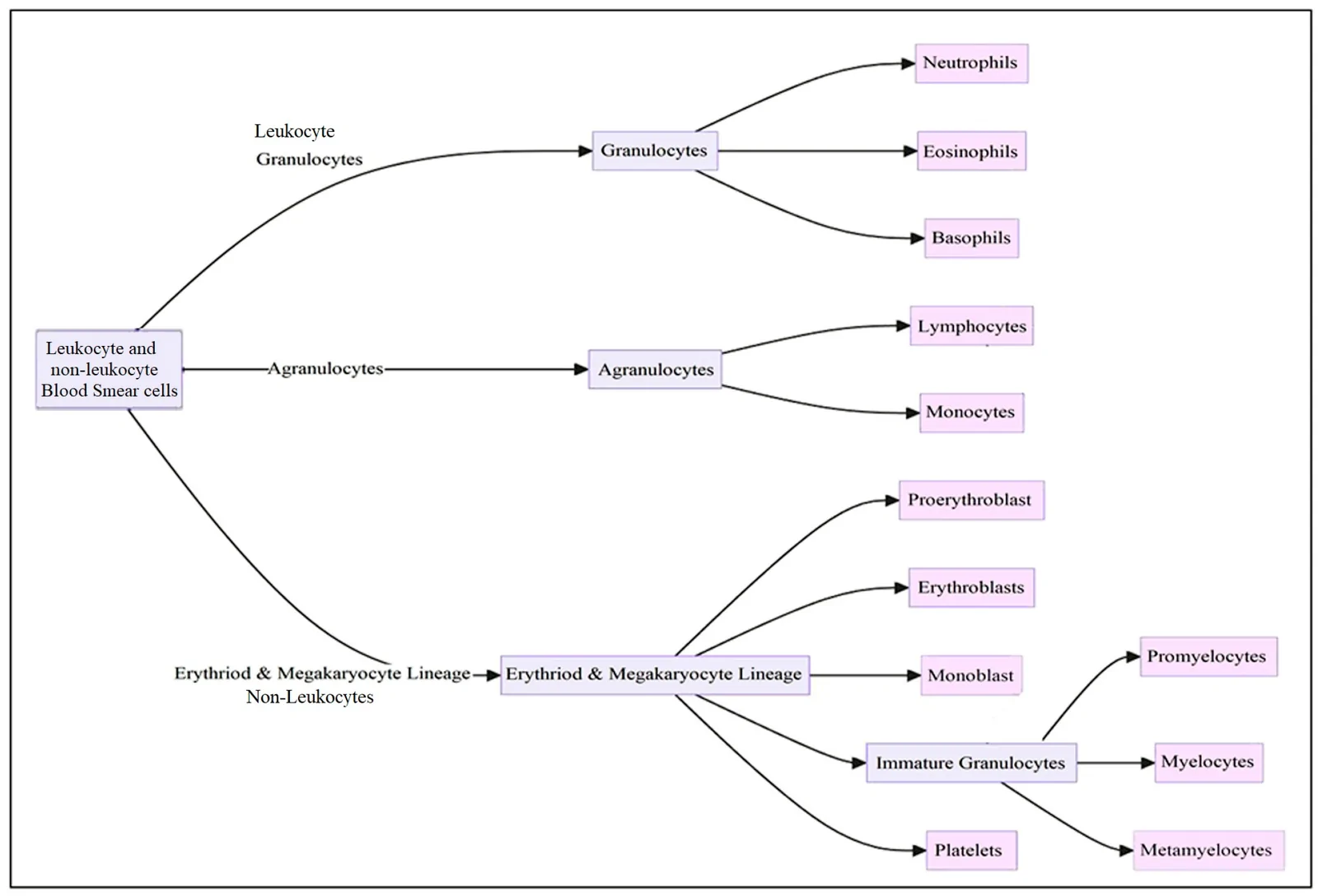
Hierarchical classification tree of 12 morphological cell types. In the first decision, the tree splits leukocytes (WBCs) from other blood cells (erythroid precursors, including erythroblasts and pro-erythroblasts, and platelets), followed by further fine-grained classification within the leukocyte population. This hierarchical approach has been selected favorably because leukemia evaluation in the clinical diagnostic setting typically mirrors generating future differential diagnostic features that distinguish between abnormal erythroid precursors or platelet abnormalities and true leukocyte populations. Its hierarchical organization naturally guides the model to learn from coarse to fine separation of morphologically similar classes, reducing potential confusion and improving the overall classification accuracy and robustness.

**Figure 2:**
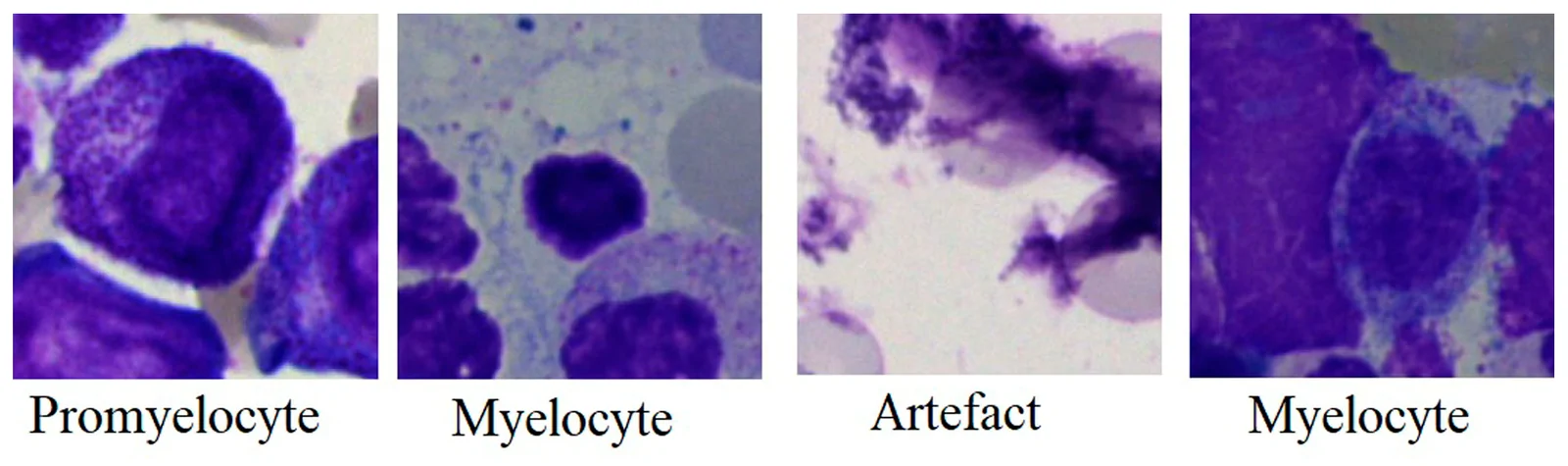
Examples of blurry, low-contrast, or unclear images were excluded from the [48] dataset during manual curation.

This study uses a combined dataset of seven cell classes from Basophil (BAS), Eosinophil (EOS), Erythroblast (EBO), Lymphocyte (LYC), Monocyte (MON), Neutrophil, and Platelets [47] and five more from [48], including Metamyelocyte (MMZ), Monoblast (MOB), Myeloblast (MYO), Pro-erythroblast (PEB), and Promyelocyte (PMO). Figure 1 shows an overview of all 12 classes. We chose 5 classes that are most relevant for leukemia classification from the Matek et al. [48] dataset, which was originally composed of 21 morphological classes with a highly imbalanced distribution and complemented the peripheral blood classes from Acevedo et al. [47]. To avoid redundancy, classes that overlapped with Acevedo et al. [47] were not included in Matek et al. [48] because the images in Acevedo et al. [47] were visually clearer and more consistently annotated. The other classes from Matek et al. [48] were discarded because of small sample size following quality filtering, poor image quality (artifacts, smudge cells), or low diagnostic priority for peripheral blood smear analysis. Images were manually checked within the 5 selected Matek classes for completeness of cell capture, focus accuracy, and the absence of overlapping cells or staining artifacts; only images that fulfilled all three criteria were selected. The complete original class distribution of the Matek et al. [48] dataset demonstrated a huge imbalance and very small sample sizes for some classes: abnormal eosinophils, 8 images; faggot cells, 47 images; smudge cells, 42 images; and immature lymphocytes, 65 images. Many of these classes also included a high proportion of out-of-focus, low-contrast duplicates, or poorly shot examples in Table 1. Figure 2 shows examples of such low-quality images that were rejected, so some of the latter classes overlapped with those in the Acevedo et al. [47] peripheral blood dataset, though the images in Acevedo et al. were visually clearer and better annotated. All images selected from the original dataset went through a strict manual curation process: blurry, low-contrast, repeating, or ambiguous images were discarded. To avoid data leakage, image-level 70-20-10 splitting was used for Acevedo et al. [47] (patient identifiers not available in the public release), and strict patient-level 70-20-10 splitting was used for Matek et al. [48] to obtain a balanced combined dataset per source by taking averages. For Matek et al. [48], all images of the same patient were allocated to a single split according to the patient identifiers provided in the original dataset metadata. No Matek patient is seen in more than one split. The images from Acevedo et al. [47] were randomly divided into train/validation/test sets at the image level. This careful selection process, as well as the exclusion of lower-quality images and curation, resulted in a high-quality, clinically relevant 12-class diabetes-friendly dataset containing 12,879 images for robust multiclass leukocyte alongside non-leukocyte classification in leukemia diagnostics. The detailed class distribution is presented in Table 2.

The dataset integrates public microscopic images from two institutions, including Hospital Clinic of Barcelona [47] and Munich Leukemia Laboratory (MLL) [48]. These two publicly available datasets were most likely subjected to different staining protocols: Wright–Giemsa for the Barcelona dataset and May–Grünwald–Giemsa for the Munich dataset. Just basic min-max intensity normalization was applied during preprocessing to minimize domain shift and prevent the model from learning site-specific or stain-specific features. This was the only way to normalize the stain without using any advanced techniques.

Note: The last 12-class dataset was built in two steps. Stage 1 (class selection): Seven classes from Acevedo et al. [47] were kept in their entirety. Five classes were chosen from Matek et al. [48] for the leukemia classification, which were relevant to the diagnosis. Stage 2 (image filtering): In the 5 selected Matek classes, images were excluded if they did not meet all three criteria: (i) incomplete capture of cells (cell partially outside the frame), (ii) out-of-focus or blurred nucleus/cytoplasm, and (iii) overlapping cells or staining artifacts. The 7 Acevedo classes were pre-validated for quality, and no image-level filtering was applied.

Acevedo et al. [47] do not include patient identifiers in the public release, but rather split the images. Matek et al. [48] split patients by patient ID to avoid data leakage.

**Figure 3:**
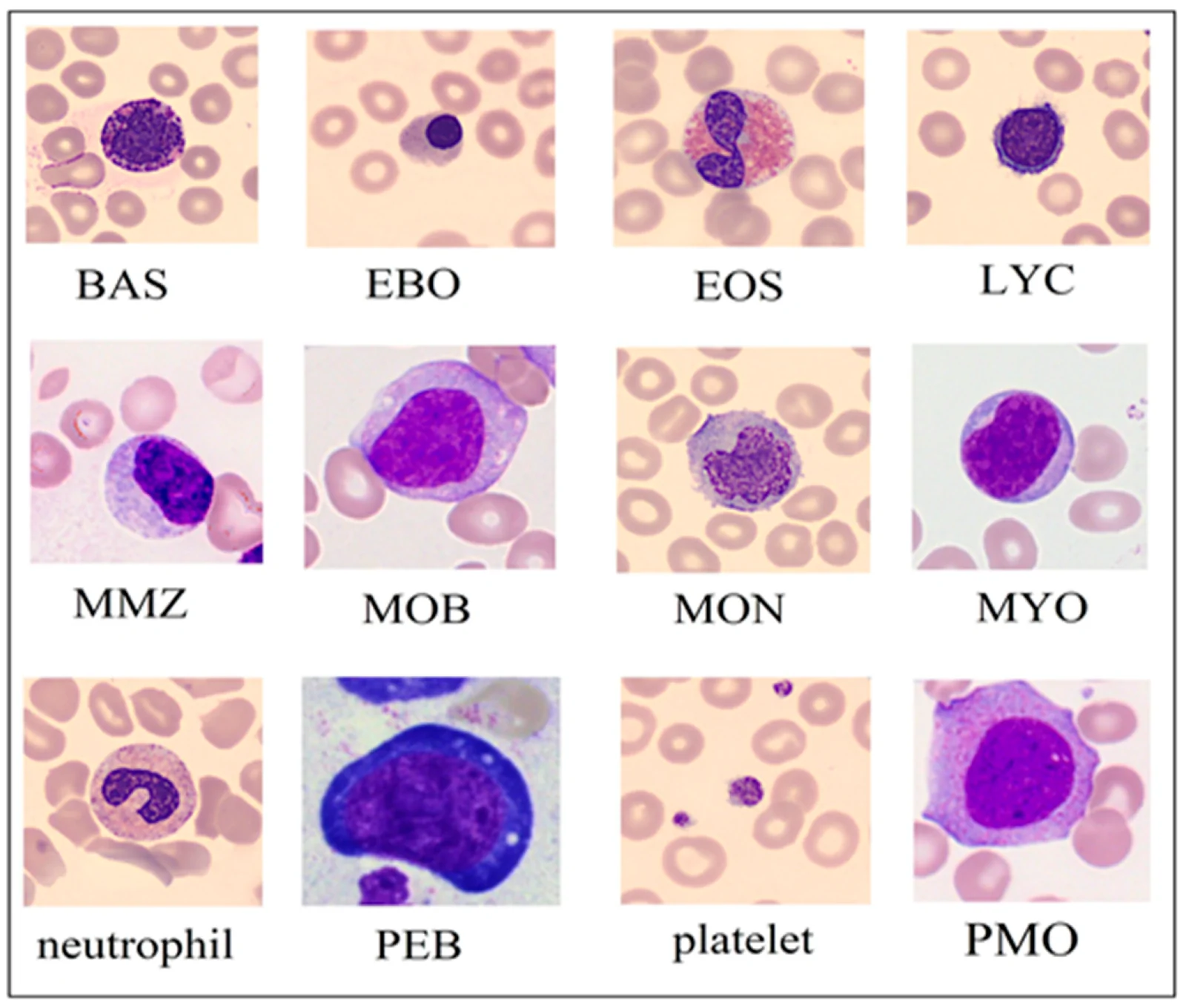
Representative images depicting each leukocyte and non-leukocyte class identified in a blood smear.

Figure 3 shows that the dataset is balanced across all 12 classes of leukocytes and well represents morphological features, such as granule density in Eosinophil and nucleus shape variation in Lymphocyte, both of which are critical for training the model.

The two datasets were deliberately combined because accurate leukemia diagnosis from peripheral blood smears requires not only mature leukocyte populations [47] but also immature and pathological cells typically observed in bone marrow [48]. Peripheral blood smear examination, which is often the initial test in investigating leukemia cases in clinical practice, commonly shows immature blasts along with abnormal erythroid precursors and megakaryocytic elements that arise from the bone marrow. We have developed a model that can generalize to not just blood cells but also to hematological cells commonly found in blood smear slides used in clinical practice, using specific classes from the two sources. This integrative strategy allows for a complete multiclass classification that more closely resembles the real-life situation of leukemia diagnosis, where distinguishing leukemic blasts from reactive or normal cells is essential.

### 3.2. Preprocessing

The preprocessing pipeline is an important step in enhancing the quality of microscopic blood smear images and normalizing them before entering them into the XHIC-Net model. This pipeline comprises several operations that account for variations in image acquisition and imaging environments, such as differences in microscope magnification, staining intensity, and environmental noise. The following outlines the process.

Normalization: The pixel intensities are normalized to a range of [0, 255], but internally normalized to [0, 1] for network training using min-max scaling: Inorm = I − Imin/Imax − Imin, where *I* is the pixel value, and *Imin* and *Imax* are the minimum and maximum intensity in an image. This operation reduces the influence of heterogeneous illumination and staining artifacts commonly observed in blood smears, thereby stabilizing feature extraction during convolution and involution.

Resized: All images were resized to 224 × 224 pixels via bilinear interpolation. This interpolates the image, assuring smooth continuity of the cell border and texture. This fixed size was selected to match the input requirements of the XHIC-Net architecture, which was inspired by networks such as VGG and DenseNet. This approach allows for high-throughput batch processing, reduces computational requirements, and preserves the morphology–phenotype continuum with specific granule detail within leukocytes.

Data Augmentation: We randomly apply transformations to the training set to improve generalization and address class imbalance issues, particularly for rare cell types such as basophils. This includes a shear range of ±20 to simulate distorted cells and a zoom range of ±20 to cover the different magnifications at which images are taken. Random horizontal or vertical flips help to cope with variations in smear orientation. In TensorFlow, for example, these augmentations are performed in real time by the ImageDataGenerator, which generates augmented samples during training. This method generates a larger dataset while avoiding overfitting through realistic variable generation.

Noise is removed by applying a Gaussian blur with a 3 × 3 kernel and a sigma of 1, followed by median filtering with a 3-pixel kernel. This approach reduces staining and scanning artifacts in the image while preserving the edge details that are essential for accurately detecting cell boundaries. No stain normalization or domain adaptation was used; only min-max intensity normalization was used to prevent domain shift between the two source datasets. This approach allows an evaluation to determine if XHIC-Net learns biologically invariant morphological features as opposed to dataset-imperative staining/acquisition artifacts.

**Figure 4:**
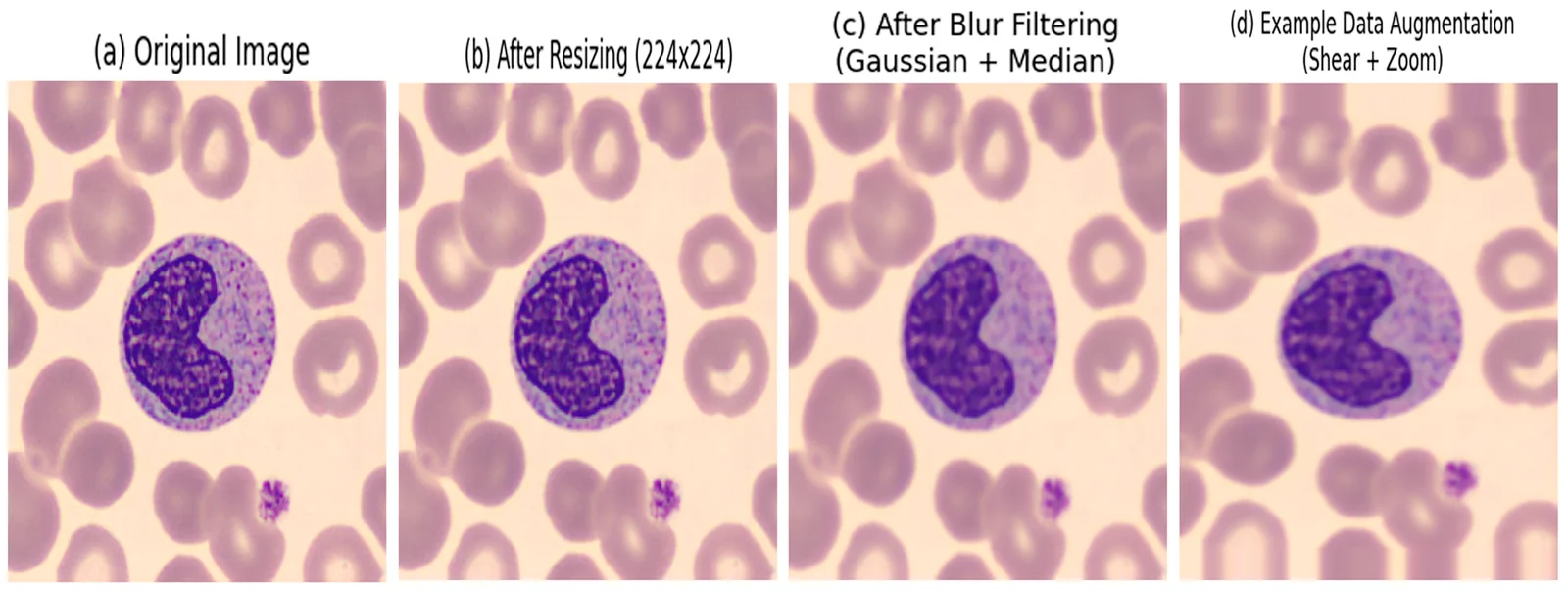
Visualization of various steps of preprocessing.

As illustrated in Figure 4, the preprocessing steps transform raw images into model-ready inputs.

### 3.3. Proposed XHIC-Net Model

**Figure 5:**
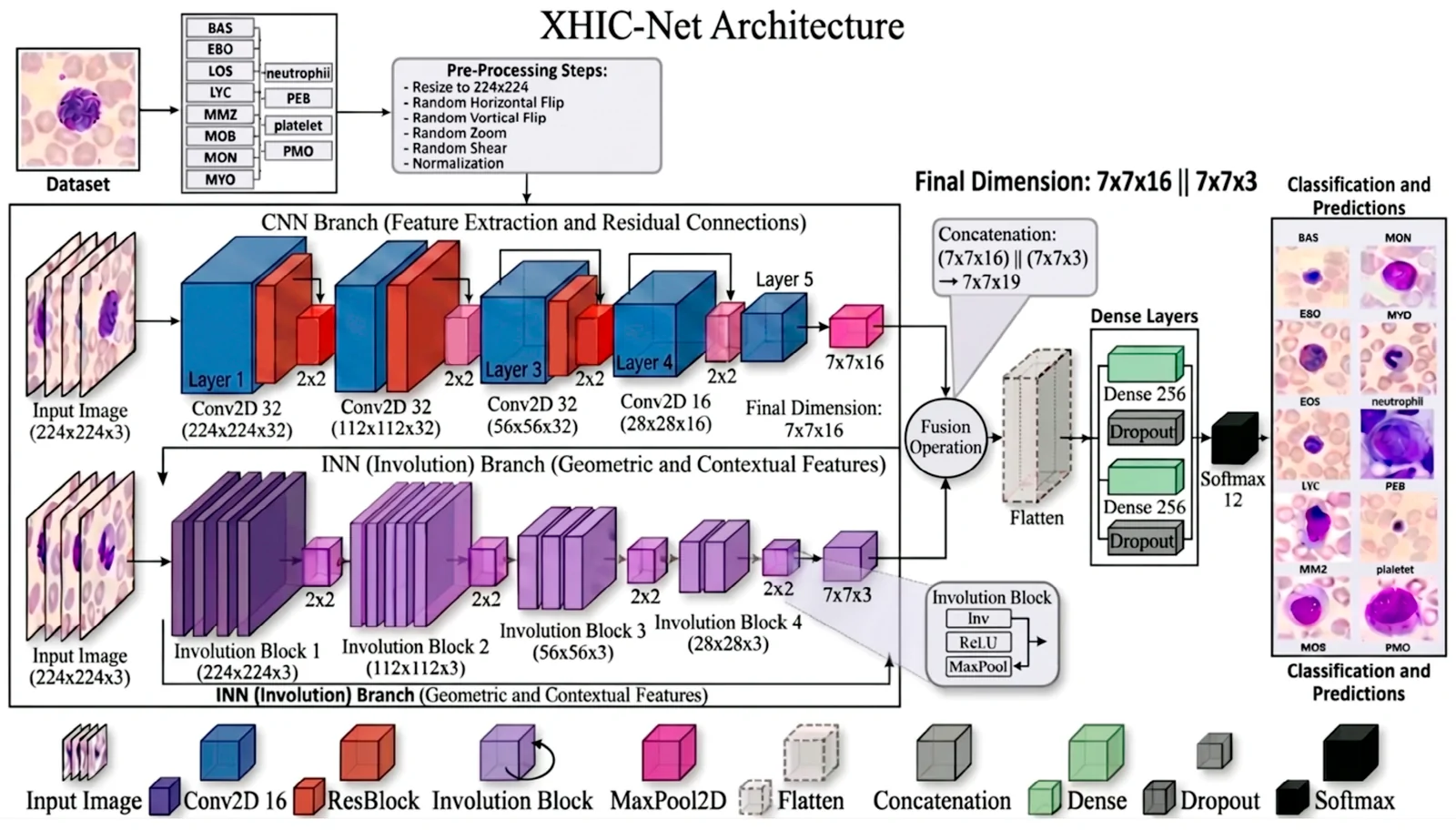
Detailed architecture of the proposed XHIC-Net model. The network consists of two parallel branches: the Involution Branch (left) and the Convolutional Residual Branch (right). Feature dimensions are shown at key stages. Outputs from both branches are concatenated before being passed to the classification head.

The XHIC-Net model architecture, as shown in Figure 5, is developed to improve blood cell classification performance. The involution operation is mathematically formulated as:

(1) $$Y_{c,i,j}=\;\sum_{k=1}^{K}\;\sum_{l=1}^{L}\;X_{c,i+k,j+l}.W_{c,k,l}(i.j)$$

where *X* is the input feature map, and *W* is the involution kernel generated adaptively for each spatial location (*i, j*). Channel *K* × *L* is the kernel size, and the summation is performed over the kernel window. In contrast to a standard convolutional kernel, which is static and shared across all spatial locations, the involution kernel *W* varies according to both spatial location and channel-wise features. This characteristic makes this operation particularly well-suited to capture subtle morphological features within blood smear images, including granule distribution and nuclear shape.

These values were chosen to optimize the balance among classification accuracy, computational efficiency, and model stability on the relatively small medical dataset used in this study. Using larger kernels greatly increases the computational cost without leading to sufficient accuracy gains. Using smaller kernels, on the other hand, results in inadequate spatial context capture. Analogously, a reduction ratio of 4 offered the best trade-off between expressiveness and efficiency, while deeper residual blocks enhanced gradient flow without inviting overfitting. This hyperparameter setting allowed XHIC-Net to surpass the stand-alone CNN and INN baselines while keeping a light architecture for clinical deployment.

The model demonstrates flexibility and can be applied to a range of biomedical image analysis tasks [49]. For XHIC-Net CNN with residual blocks and an INN [14], this combination reduces parameter count while preserving high accuracy. The CNN layers with residual blocks function alongside the INN, which forms the core of XHIC-Net. The INN enhances discriminative capability with fewer parameters when the number of classes is limited; therefore, optimal feature extraction is achieved by restricting the number of classes.

The input is processed by two branches: the involution branch, which focuses on spatial adaptive features, and the CNN branch, which uses a residual block to extract hierarchical patterns. These outputs are then concatenated at the feature level, enabling the fusion of complementary information before classification.

An essential part of representation learning in the proposed method is an involution operation followed by feature extractors that generate respective dynamic kernels to capture spatial context. Subsequently, CNN residuals are aggregated to embody the multi-scale hierarchical characteristics. In particular, the early layers detect edges and nuclei, the mid-layers learn texture motifs, and the late layers summarize information for discriminative purposes. By using this hierarchy, we can minimize feature redundancy, leading to more accurate leukocyte recognition and non-leukocyte blood smear cell types. A detailed architectural diagram with feature dimensions and fusion operations is presented in Figure 5.

Table 3 shows the layout of our architecture, XHIC-Net. It is composed of two parallel branches, with one being a convolutional branch consisting of residual blocks organized in a pyramid fashion for hierarchical feature extraction and the other an involution branch that captures spatially adaptive features. The outputs from these two branches are concatenated and fed into a classification head that predicts one of 12 blood cell classes. This hybrid design enables XHIC-Net to maintain high accuracy but with much fewer parameters in contrast to traditional CNN models.

The proposed model combines an involution branch and a convolutional neural network (CNN) branch with residual connections for image classification. The input image is denoted by *X* ∈ ℝ^H × W × C, where H = 224, W = 224, and C = 3 (RGB channels). The model outputs class probability for N = 12 classes.

The involution layer generates spatially adaptive kernels, productized kernels in convolutions. It processes input $X\in R^{H\times W\times C}$ to produce output $Y\in R^{H\times W\times C}$, where *H′* = ⌊*H*/*S*⌋, *W*′ = ⌊*W*/*S*⌋, and S is the stride.

• Stride Layer: Downsamples the input if S > 1 using average pooling:
  (2) $$X_{down}=\{\begin{array}{c} {Avgpool}_{s\times s}\\ X, \end{array}(X),\;\mathrm{if}\;S\;>\;1$$
  where $X_{down}\in R^{H\prime \times W\prime \times C}$
• Kernel Generation: A neural network generates dynamic kernels:
  (3) $$KernelGen={Conv2D}_{1\times 1}^{K^{2}G}\text{◦}ReLU\;◦\;BN\;◦{Conv2D}_{1\times 1}^{C/R}$$
  where K is the kernel size, G is the group number, R is the reduction ratio, and:
  (4) $$K\;=\;Reshape(KernelGen(X_{Down})),\;K\in R^{H\prime \times W^{\prime }K^{2}\times 1\times G}$$
  This generates spatially adaptive kernels for each location (i, j).
• Patch Extraction: Extracts K × K patches from the input:
  (5) $$P=ExtractPatches\left(X,size=\left[K,\;K\right],stride=S,\;padding=SAME\right)$$
  (6) $$P\prime =Reshape\left(P\right),P^{\prime }\in \;R^{H^{\prime }P^{\prime }\in \;R\;H^{\prime }\times W^{\prime }\times K2\times \left(\frac{C}{G}\right)\times G}$$
  Channels for efficient processing of group patches.
• Involution Operation: Multiplies kernels with patches and sums:
  (7) $$Y\prime \;(i,\;j,\;:,\;:,\;g)=K\left(i,\;j,\;:,\;:,\;g\right)\cdot \;P\prime \left(i,\;j,\;:,\;:,\;g\right)$$
  (8) $$Y\prime \prime (i,\;j,\;:,\;g)=\sum_{k=1}^{k^{2}}Y\prime (i,\;j,\;k,\;:,\;g)$$
  (9) $$Y=Reshape(Y\prime \prime ),\;Y\;\in R^{H\prime \times W\prime \times C}$$

### 3.4. The Proposed CNN Architecture

The proposed CNN architecture integrates residual blocks to enhance feature extraction for leukocyte classification. Incorporating shortcut connections within these residual blocks addresses the vanishing gradient problem and improves gradient flow during backpropagation. This design enables the model to learn complex representations, resulting in performance comparable to that of significantly deeper networks.

It consists of an architecture comprising a stack of convolutional layers (ReLU). The CNN branch comprises five standard layers, followed by batch normalization and a 3 × 3 ReLU activation. This gives a total of around 14 effective layers, because residual blocks (consisting of two convolutional layers each) are used over them. The chosen depth aimed to strike a balance between more complex models and gradient flow; architectures with 14 layers failed to reach comparable accuracy, while networks deeper than this were expected to overfit, although this had not been empirically tested. To improve feature representation, each residual block includes two 3 × 3 convolutional layers, ReLU activations, and shortcut connections. Each block includes a 2 × 2 pooling layer, which drastically reduces spatial dimensions and computational cost. The final CNN feature is combined with the INN output to collect other salient features in context. These features are then concatenated, flattened, and fed through dense layers with ReLU activation and a 20% dropout rate until convergence to mitigate overfitting. A dense layer with a SoftMax activation provides likelihood estimates for 12 leukocyte subtypes. This deep architectural design uses convolutional and residual learning to create a deeper model that improves classification.

**Figure 6:**
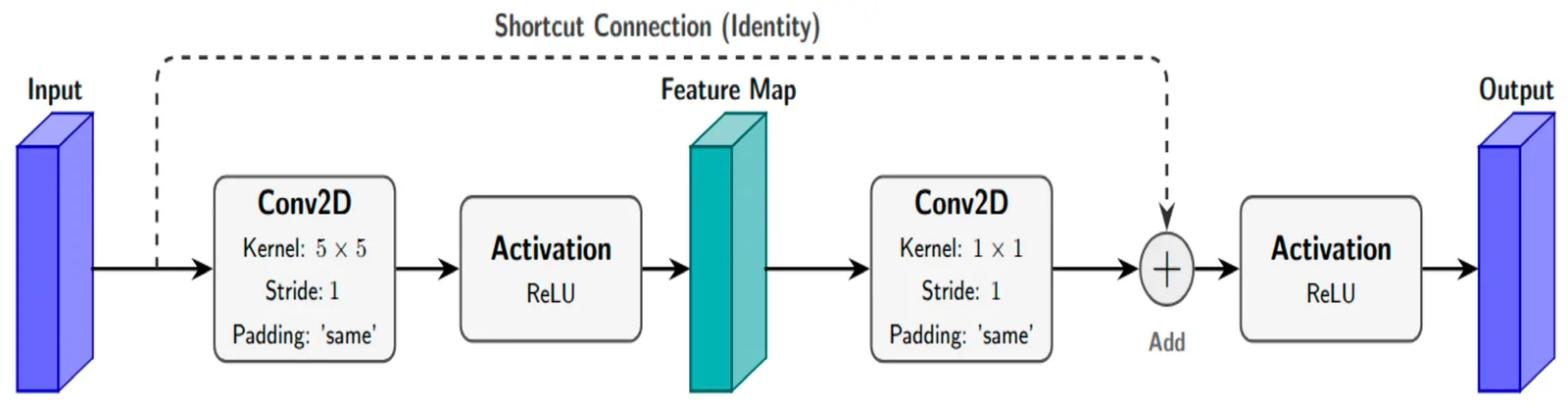
Demonstrated structure of the residual CNN component.

A complete representation of the residual block structure is illustrated in Figure 6, where data flows from the Conv2D layer to a residual module. In the process, the input pixels are convolved with a 5 × 5 kernel, followed by a 1 × 1 kernel convolution (with stride one and ReLU activated) and eventually a max-pooling layer. As illustrated in Figure 5, it performs efficient feature extraction, retaining essential information while leveraging shortcuts across layers to minimize spatial dimensions.

### 3.5. The Involution Neural Network (INN)

While the INN is a relatively well-known architecture, its implementation and integration in the current framework are new. The INN is a context in which we hypothesize a feature-extraction mechanism that captures spatially adaptive representations in leukocyte images. Regular convolutional layers have spatially invariant kernels that assume the same kind of structure across the entire image. This approach is beneficial when dealing with general vision tasks, but it cannot model localized morphological changes found in leukocytes and blood smear images. The suggested framework takes advantage of the spatial adaptivity in INN and thus has increased sensitivity for more subtle morphological differences within leukocyte subpopulations. The INN also alleviates parameter redundancy by a factor of about 40–50%, while enabling better discrimination, thus encouraging local and context feature learning. Unlike conventional CNNs, which cannot recognize spatial uniformity and exist in a constant domain of uncertainty, integration more effectively manages the variable morphologies present in the unique landscapes of medically relevant peripherals used in leukocyte analysis, preventing them from identifying morphological differences between various types of leukemia cells. Consequently, the framework abstracts local patterns and vice versa, indicating contextual relationships. In comparison, convolution applies a homogeneous kernel at all spatial locations and may fail to capture this subtle discriminant information [50].

Another significant advantage is channel invariance. In conventional convolutional operations, different kernels are applied independently to each channel, leading to redundancies. The INN [14] addresses this by using the same kernel across all channels, thereby reducing computational complexity and minimizing inter-channel redundancy. This method also efficiently processes multi-trimmed data, such as RGB microscopic images [50]. Furthermore, INN [14] captures extensive spatial context by adaptively accumulating weights across various locations and prioritizing more informative spatial features. These adaptive penalization weights allow the model to focus on critical leukocyte features, which enhances classification performance. The INN further improves discrimination by learning distinct adaptive kernels for each spatial location, while using fewer parameters and sharing channels, resulting in 50% fewer parameters than standard CNNs. In a 12-class configuration, this approach achieves approximately 98% feature-extraction accuracy (as verified on a preliminary set of cases not used for training) when identifying class-specific morphologies, such as irregular nuclei in myeloblasts.

Multiple strategies have been proposed to enhance the performance of the INN within XHIC-Net. The use of adaptive kernel sizes, such as dynamically alternating between 3 × 3 and 5 × 5 kernels according to spatial complexity, increases flexibility in capturing diverse leukocyte morphologies. Preliminary results demonstrate an improvement in accuracy of approximately 2–3% [51]. Additionally, introducing a batch normalization layer after each involution step, rather than only after sequential layers, stabilizes training by reducing internal covariate shift and accelerates convergence by up to 15% [51]. Furthermore, generalizing the reduction ratio parameter to a range allows for finer control over computational efficiency. This method is particularly advantageous for optimizing performance on large datasets, such as the leukocyte dataset [50], which comprises 12,879 images utilized in this study. Collectively, these enhancements are anticipated to improve the model’s robustness, effectiveness, and generalizability for practical medical applications.

**Figure 7:**
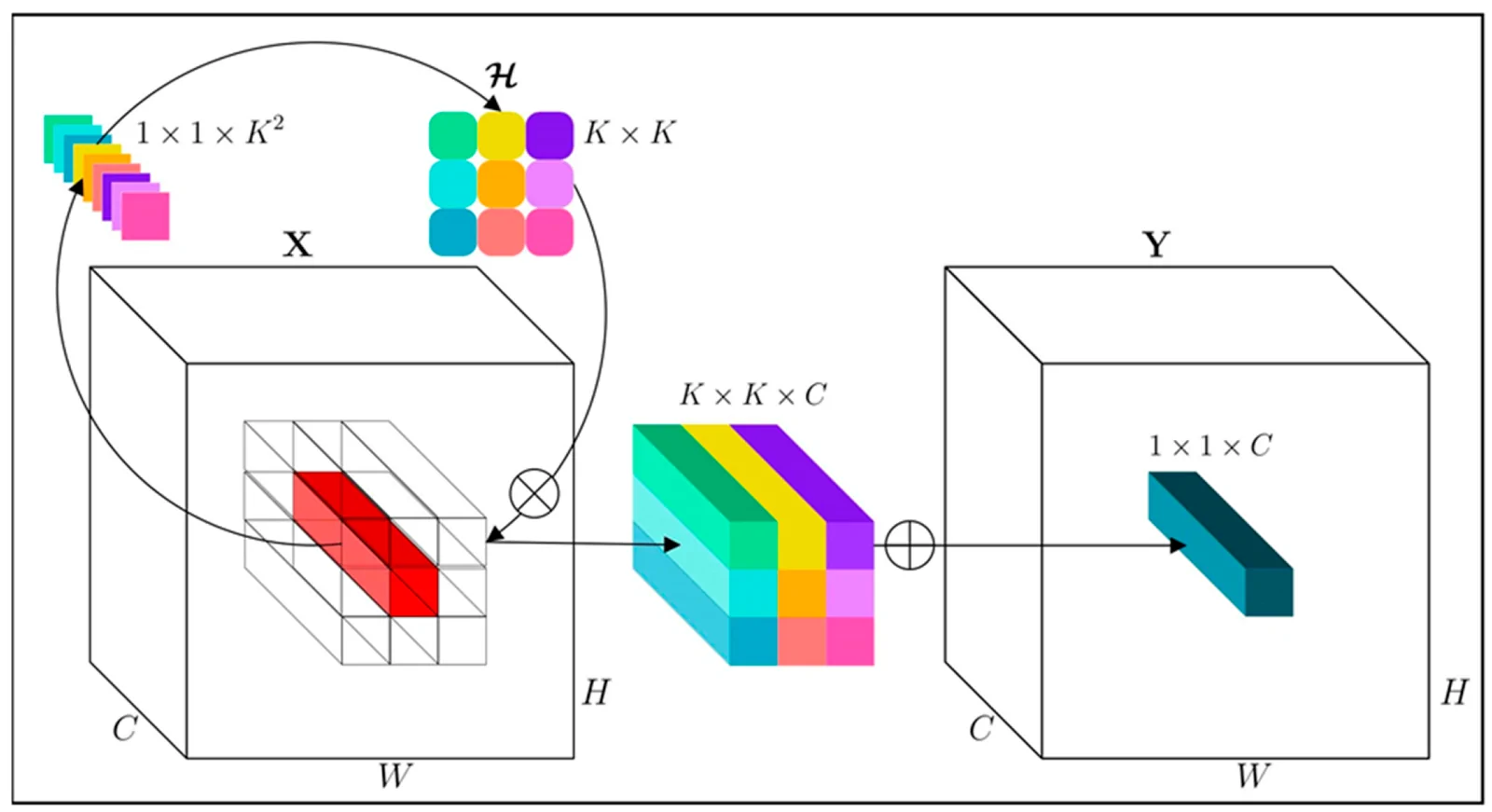
The architecture of the Involution Neural Network (INN) is illustrated.

While the INN architecture uses 3 × 3 kernels, conventional convolution uses 1 × 3 and 3 × 1 kernels with a stride of 1 × 1. Involution followed by data normalization solves the problems of gradient dissipation and internal covariate shift. Figure 7 shows that, after convolution, a 2 × 2 max-pooling layer is applied, and the ReLU (Rectified Linear Unit) activation function improves the model’s nonlinearity. ReLU and data normalization facilitate stochastic gradient descent in XHIC-Net by smoothing the input distribution at intermediate layers. This results in fewer weight updates being required during training.

Figure 5 presents the INN [14] architecture and its data processing pipeline. In this context, ‘involved’ denotes the application of the involution operation, in which active kernels process input patches to aggregate features. The figure shows data flow—input, kernel generation, patch-wise multiplication, and summation that promotes spatial adaptation while using fixed filters to apply convolution. The pipeline contains a 3 × 3 involution kernel with a stride of 1, data splitting, normalization, and ReLU activation before addition. The rest of the visualization proceeds to the 2 × 2 max-pooling layer, showing how spatial dimensions get narrowed down as the INN ingests and processes information.

Table 4 presents the operational flow of INN [14], which takes channel count, kernel size, stride, and reduction ratio as input parameters. During the build phase, a height × width matrix is calculated and multiplied using a stride layer with an activation function that allows for non-linearity. On the other hand, the sequential module consists of batch normalization and two 3 × 3 convolutional layers with this network kernel size and group settings. The reshape operation updates the kernel to match the input dimensions. In the forward pass, patches are extracted from the input data, multiplied by the kernel, and reshaped to their original configuration. This process, as outlined in the algorithm, facilitates efficient feature extraction and context summarization. Normalization and activation operations contribute to stable training. Applying normalization at each stage, along with adaptive kernel scaling, is expected to improve this process further and enhance performance in vision-based recognition tasks [51].

In Table 4, we present a summary of the operation flow for INN with respect to parameterized channel count, kernel size, stride, and reduction ratio. Layer 1: Model layering of stride, tracking, and tracing with the addition of a nonlinearity inherent through an activation function through a width × height matrix. We have two 3 × 3 convolutional layers, followed by batches in which the kernel size and group size are used. In this step, we reshape the kernel to match the input shape. During the forward pass, patches of the input data are extracted and multiplied element-wise by the kernel. Then, they are reshaped back to their original form. The algorithm provides detailed information on how such a process will work, helping us to extract the right features and summarize effectively. Normalization and activation functions guide and help train our model. The involution layer has time complexity *O(HWCK^2/G)*, where H and W denote the height and width, C is the channel size, K is the kernel size, and G is the group size. With K = 3 and G = 16, this approach is approximately twice as fast as standard convolution (O(HW*C^2)), enabling real-time inference at less than 10 ms per image on a GTX 1660.

The complete algorithmic workflow for the involution layer is implemented as follows: (1) Input *X ∈ R^*(*H × W × C*) is downsampled via average pooling if stride *S > 1*; (2) kernel generation network produces *K ∈ R^*(*H′* × *K*^2^ × 1 × *G*) via two 1 × 1 convolutions with BatchNorm and ReLU; (3) patches *P ∈ R^*(*H′* × *W′* × *K*^2^ × (*C*/*G*) × *G*) are extracted and reshaped; and (4) element-wise multiplication and summation over K^2^ yields output *Y ∈ R^*(*H′* × *W′* × *C*). Group size G = 16, reduction ratio R = 2, and kernel size K = 7 were used. This implementation follows the TensorFlow/Keras framework and is fully specified in the released code.

### 3.6. Involution Branch

The involution branch applies five involution layers, each followed by ReLU and max-pooling. For the n-th layer:

(10) $$X_{inv,n}=\;{MaxPool}_{2\times 2}(ReLU(Involution(X_{inv,n-1})))$$

where $X_{inv,0}$ = X and parameters are C = 3, G = 1, K = 3, S = 1, R = 2.

The final output is $X_{inv,5\;}$ ∈ $R^{7\times 7\times 3}$. This branch extracts spatially adaptive features with progressive spatial reduction.

### 3.7. Residual Block

The residual block mitigates vanishing gradients by adding a shortcut connection:

(11) $$X_{out}=ReLU(X\;+\;f(X))$$

where

(12) $$f(X)={{Conv2D}^{F}}_{1\times 1}(ReLU({{Conv2D}^{F}}_{5\times 5}\;(X)))$$

And F is the number of filters. The block preserves input dimensions if C = F; otherwise, a 1 × 1 convolution is applied to adjust the shortcut.

Each residual block in Figure 4 includes two 3 × 3 convolutional layers to refine features and rectified linear unit (ReLU) activations for non-linear transformation (identity shortcut connection). The first block is designed to undertake the basic edge detection. It blocks 2–4 build progressive feature hierarchies to retain key information, including cellular granules, while reducing gradient loss across multiple layers.

### 3.8. CNN Block

The CNN branch starts with a convolution, followed by four residual blocks and additional convolutions, each with max-pooling:

(13) $$X_{cnn,0}={{Conv2D}^{32}}_{3\times 3}(X)$$

(14) $$X_{cnn,n}={MaxPool}_{2\times 2}\;{(ResidualBlock({Conv2D}_{3\times 3}}^{F_{n}}\left(\;X_{cnn,n}-1))\right)\;n=1,\;2,\;3,$$

(15) $$X_{cnn,4}={MaxPool}_{2\times 2}(ResidualBlock({{Conv2D}_{3\times 3}}^{F_{n}}(X_{cnn,3})))$$

(16) $$X_{cnn,5}={MaxPool}_{2\times 2}({{Conv2D}^{16}}_{3\times 3\;}\;(X_{cnn,4}))$$

where $F_{n}$ = 32 for *n* = 1, 2, 3, and $F_{4}$ = 16. The output is $X_{cnn,5}$ ∈$R^{7\times 7\times 16\;}$

### 3.9. Concatenation and Classification

The outputs of both branches are concatenated and processed through dense layers:

(17) $$X_{concat}[X_{inv,5},X_{cnn,5}],\;X_{concat\;}\in \;R^{7\times 7\times 19}$$

(18) $$X_{flat}=Flatten\;\left(X_{concat}\right),\;X_{flat}\in \;R^{7\cdot 7\cdot 19}$$

(19) $$X^{1}=ReLU(W1Xflat+b1),\;W_{1\;}\in \;R^{931\times 256}$$

(20) $$X_{2}={Dropout}_{0.2\;}\left(X1\right),$$

(21) $$X_{3}=ReLU(W_{2}X_{2}+b_{2}),W_{2}\;\in \;R^{256\times 256},$$

(22) $$X_{4}={Dropout}_{0.2}(X3),$$

(23) $$Y=Softmax(W_{3\;}X_{4}+b_{3}),\;W_{3\;\in \;}R^{256\times 12},\;Y\;\in \;R^{12}$$

### 3.10. The Statistical Methods for Pairwise

The statistical methods for Pairwise Model Comparison are presented. McNemar’s test is used to test for statistically significant differences in performance between XHIC-Net 3 and other models, along with Cohen’s h effect size. As per-sample prediction labels are not available, we first estimate conservative disagreement labels directly from the reported overall accuracy. If two models, acc1 and acc2, have overall accuracy on n test samples, the number of samples correctly classified by each model is estimated as:

(24) $$C_{1}=round\left({acc}_{1}.\;n\right),\;C_{2}=round({acc}_{2}.n)$$

where $c_{1}$ and $c_{2}$ represent the total correct prediction by model 1 and model 2, respectively. The overlapping connect prediction and the exclusive disagreements are then derived:

(25) $$both=min(c_{1,\;C_{2}}),b=max(0,c_{1-both}),c=max(0,c_{2-both})$$

Here, both means the number of samples that are classified correctly by both models; b denotes the number of samples that are classified correctly by Model 1 and incorrectly by Model 2; and c denotes the number of samples that are classified incorrectly by Model 1 and correctly by Model 2. This formulation gives a conservative lower bound on the actual disagreement counts if individual prediction labels are not available. McNemar’s test statistic with continuity correction is calculated as:

(26) $$X^{2}=\;\frac{(\left|b-c\right|-1)2}{b+c}$$

where b and c are the off-diagonal disagreement counts from the contingency table of paired predictions. The term ∣b − c∣ reflects the absolute degree of asymmetry of disagreement, the correction term −1 gives a conservative estimate for small sample sizes, and the denominator b + c is the number of pairs of disagreement. If b + c = 0, the test statistic is undefined and *p* = 1.0 is returned. The statistical significance of the observed disagreement asymmetry is determined by:

(27) $$p=1-F_{x2}(X^{2},\;df=1)$$

where Fχ2 is the cumulative distribution function of the chi-squared distribution with one degree of freedom (df = 1). This *p*-value is used to test the null hypothesis H0: b = c (both models have the same error rates) against the two-tailed alternative H1: b = c. A small *p*-value means that the difference in performance between the two models is not likely to be due to chance. Effect size measures the actual size of the difference, whereas statistical significance determines whether a difference exists. For comparing two proportions (accuracies), Cohen’s h is defined as:

(28) $$h\;=\;2(arcsin\sqrt{p1-arcsin\sqrt{p2}})$$

p1 and p2 are the accuracy proportions of the reference model (XHIC-Net 3) and comparison model, respectively. The arcsin⋅ transformation normalizes the variance of proportion estimates, and the 2 scales the difference to standard effect size conventions. The sign of h denotes direction: positive h means that the reference model is favored, whereas negative h means that the comparison model is favored. The 95% confidence interval is built as:

(29) $$SE=\;\sqrt{\frac{1}{4n}}\;,\;{CI}_{95\%\;}=h\pm z0.975\;.\;SE$$

The standard error of Cohen’s h, the critical value of the standard normal distribution at the 97.5% percentile (z0.975 ≈ 1.96), and n is the number of test samples. The width of the intervals represents the uncertainty of the estimates, and intervals that do not contain zero represent statistically significant effect sizes at α = 0.05.

## 4. Results

This section may be divided into experimental setup and results. It should provide concise and precise information.

### 4.1. Experimental Setup

This section presents an experiment conducted to evaluate the proposed XHIC-Net model. To address the class imbalance problem, the dataset was balanced before testing. Notably, 70% of the data was used for training, 10% for validation, and 20% for testing. A portion of the test data was then selected for verification. This ensured that the model was trained on a representative sample and properly validated. All experiments were performed using TensorFlow and Keras on a system configured with a Core i7 3770 CPU, Nvidia RTX 2060 Super GPU, and 20 GB of RAM. We trained using the ADAM optimizer that had a fixed learning rate of 0.0001 with no decay schedule. Early stopping on validation loss and patience = 10 epochs restored best weights. The dataset was balanced, so no class weighting was applied. He normal initialization was applied for all convolution and dense layers. Regularization was done using dropout only (rate = 0.2); no L2 weight decay was applied.

### 4.2. Performance Metrics

The performance of XHIC-Net is assessed using several metrics, including overall accuracy, precision, recall (sensitivity) and Cohen’s Kappa (k). These evaluation parameters are calculated based on a multi-class confusion matrix. Equations (30)–(33) summarize the definitions of these assessment metrics. For each experiment, the multi-class confusion matrix is utilized to determine the true positive (TP), true negative (TN), false positive (FP), and false negative (FN) rates.

(30) $$Accuracy=\frac{TN+TN}{TP+TN+FP+FN}$$

(31) $$Precision=\frac{TN}{TP+FP}$$

(32) $$Recall=\frac{TP}{TP+FN}$$

(33) $$k=\frac{p_{o}-p_{e}}{1-p_{e}}$$

### 4.3. Conduct All Experiments

Comparative analysis presented in Table 5 of classification metrics for various deep learning architectures shows significant differences in performance for leaf disease recognition tasks. The proposed XHIC-Net 3 architecture achieves state-of-the-art results, with an accuracy of 98.88% (95% CI: 98.45–99.31%), a minimum top-1 error rate of 1.12%, and a standard deviation of 0.0073, which demonstrates excellent consistency across test folds. This performance is significantly higher than that of traditional convolutional networks like VGG16 (97.10%) and DenseNet121 (95.98%), and more efficient networks like EfficientNetB0 (94.60%). In particular, XHIC-Net 3 outperforms the next two best models, EfficientNetV2S and MobileNetV3Large, by 0.48 percentage points with an accuracy of 98.40%. The Cohen’s kappa of 0.9877 for XHIC-Net 3 indicates almost perfect inter-rater reliability, further supporting the reliability of predictions. The XHIC-Net variants show a progressive improvement from XHIC-Net 1 (97.41%) to XHIC-Net 4 (98.44%), with XHIC-Net 3 being the best-performing configuration. In comparison, XHIC-Net 5 (98.10%) shows some degradation, indicating possible over-parameterization effects. With an accuracy of just 90.06%, the INN baseline underscores the importance of the proposed symbiotic convolution mechanism in enhancing feature discriminability for fine-grained plant pathology classification.

**Table 5:** Performance outcomes for various residual block variants and comparative models.

Method	Precision	Recall	F1-Score	Accuracy with CI	Top-1 Error	Std Deviation	Cohen’s Kappa
DenseNet121	96.06% (89.53%, 102.59%)	95.98% (90.52%, 101.45%)	0.9599 (0.9088, 1.0111)	95.98% (95.18%, 96.78%)	4.02%	0.0261	0.9561
DenseNet169	97.18% (93.21%, 101.14%)	97.15% (94.43%, 99.87%)	0.9715 (0.9445, 0.9985)	97.15% (96.47%, 97.83%)	2.85%	0.0138	0.9689
DenseNet201	97.47% (94.18%, 100.76%)	97.45% (91.66%, 103.24%)	0.9744 (0.9389, 1.0098)	97.45% (96.81%, 98.09%)	2.55%	0.0181	0.9722
EfficientNetB0	95.80% (78.07%, 113.53%)	94.58% (70.83%, 118.34%)	0.9444 (0.7782, 1.1105)	94.60% (93.67%, 95.52%)	5.40%	0.0848	0.9410
EfficientNetV2S	98.48% (95.30%, 102.70%)	98.40% (95.64%, 102.29%)	0.9841 (0.9656, 1.0137)	98.40% (97.55%, 98.38%)	1.54%	0.0143	0.9825
MobileNet	97.19% (92.92%, 101.46%)	97.15% (92.07%, 102.23%)	0.9715 (0.9372, 1.0057)	97.15% (96.47%, 97.83%)	2.85%	0.0175	0.9689
MobileNetV2	97.51% (93.89%, 101.12%)	97.50% (93.78%, 101.21%)	0.9750 (0.9423, 1.0076)	97.49% (96.86%, 98.13%)	2.51%	0.0167	0.9726
MobileNetV3Large	98.52% (92.40%, 104.65%)	98.40% (94.10%, 102.70%)	0.9842 (0.9518, 1.0165)	98.40% (97.89%, 98.91%)	1.60%	0.0165	0.9825
MobileNetV3Small	98.44% (93.63%, 103.25%)	98.36% (94.10%, 102.62%)	0.9836 (0.9570, 1.0103)	98.36% (97.84%, 98.88%)	1.64%	0.0136	0.9821
VGG16	97.28% (90.21%, 104.34%)	97.11% (87.83%, 106.39%)	0.9708 (0.9169, 1.0248)	97.10% (96.42%, 97.79%)	2.90%	0.0275	0.9684
Based CNN	97.46% (94.56%, 100.36%	97.45% (94.04%, 100.85%)	0.9745 (0.9494, 0.9995)	97.45% (96.81%, 98.09%)	2.55%	0.0128	0.9722
CNN-INN	97.83% (93.68%, 100.79%)	97.45% (91.80%, 102.58%)	0.9718 (0.9438, 0.9998)	97.19% (96.52%, 97.86%)	2.81%	0.0143	0.9693
XHIC-Net 1	97.45% (93.23%, 101.67%)	97.40% (92.28%, 102.53%)	0.9740 (0.9407, 1.0073)	97.41% (96.76%, 98.05%)	2.59%	0.0170	0.9717
XHIC-Net 2	98.19% (94.92%, 101.47%)	98.18% (95.21%, 101.15%)	0.9818 (0.9544, 1.0093)	98.18% (97.64%, 98.73%)	1.82%	0.0140	0.9802
XHIC-Net 3	98.89% (96.71%, 101.06%)	98.87% (96.66%, 101.09%)	0.9887 (0.9743, 1.0031)	98.88% (98.45%, 99.31%)	1.12%	0.0073	0.9877
XHIC-Net 4	98.46% (96.16%, 100.75%)	98.44% (95.80%, 101.08%)	0.9844 (0.9664, 1.0025)	98.44% (97.94%, 98.95%)	1.56%	0.0092	0.9830
XHIC-Net 5	98.13% (95.08%, 101.18%)	98.10% (94.50%, 101.69%)	0.9809 (0.9627, 0.9991)	98.10% (97.54%, 98.65%)	1.90%	0.0093	0.9792
INN	90.15% (75.83%, 104.46%)	90.05% (75.56%, 104.54%)	0.9004 (0.7640, 1.0369)	90.06% (88.84%, 91.28%)	9.94%	0.0696	0.8915

A detailed efficiency study was carried out to evaluate the practical feasibility of the proposed architectures against the baseline architectures, including the number of parameters, storage size, training convergence time, inference latency per image, throughput, and GPU memory usage, as shown in Table 6. The XHIC-Net family is very compact, ranging from 1.49 MB (XHIC-Net 1) to 2.19 MB (XHIC-Net 5), which is only 2.1–3.0% of the DenseNet201 footprint (72.0 MB) and 0.6–0.9% of the EfficientNetV2S footprint (234 MB). The efficiency is also remarkable, as XHIC-Net 3 requires 414,168 parameters, while EfficientNetV2S requires 20.3 million parameters, a reduction of more than 98%. Even with this extreme compression, XHIC-Net 3 still has an inference time of 5.56 ms per image and a throughput of 180.0 FPS, comparable to lightweight baselines like MobileNet (193.6 FPS) and MobileNetV2 (193.3 FPS). The training time per epoch for XHIC-Net 3 (66.26 s) is longer than the Base CNN (26.14 s) because of the feature aggregation mechanism of the involution, but this is justified by the significant accuracy improvements shown in the performance evaluation. The total GPU memory usage for all the XHIC-Net variants is less than 7.3 GB, which is comparable to or less than most baseline architectures, thus verifying the applicability of the proposed models for edge-computing deployment in resource-constrained agricultural monitoring systems.

The stratified bootstrap resampling with replacement was performed at the sample level to maintain the class distribution per fold to calculate the confidence intervals for accuracy, precision, recall, and Cohen’s Kappa, as shown in Table 7. McNemar’s exact test was used for pairwise comparisons of models, with the 2313 predictions in the test set compared between XHIC-Net 3 and each baseline. Cohen’s h was used to quantify effect sizes, and 95% CIs were computed.

The standalone INN baseline performed significantly worse, with an accuracy of 88.93%, mainly due to the difficulty in discriminating between morphologically similar classes like myeloblasts (MYO), monoblasts (MOB), and immature erythroid precursors (PEB and EBO), which necessitate fine-grained discrimination of nuclear shape, chromatin patterns, and granule distribution. This limitation is caused by the fact that pure involution operations are efficient in capturing spatially adaptive features but do not provide the hierarchical multi-scale feature extraction that is offered by the convolutional residual branch of XHIC-Net.

The INN hyperparameters (kernel size K = 3, groups G = 16, reduction ratio R = 2) were adopted from the original Involution paper [14] and subsequently validated through our ablation experiments presented in Section 4 Table 8 and Table 9 confirming they provide an effective balance between computational efficiency and accuracy for this medical imaging task.

**Figure 8:**
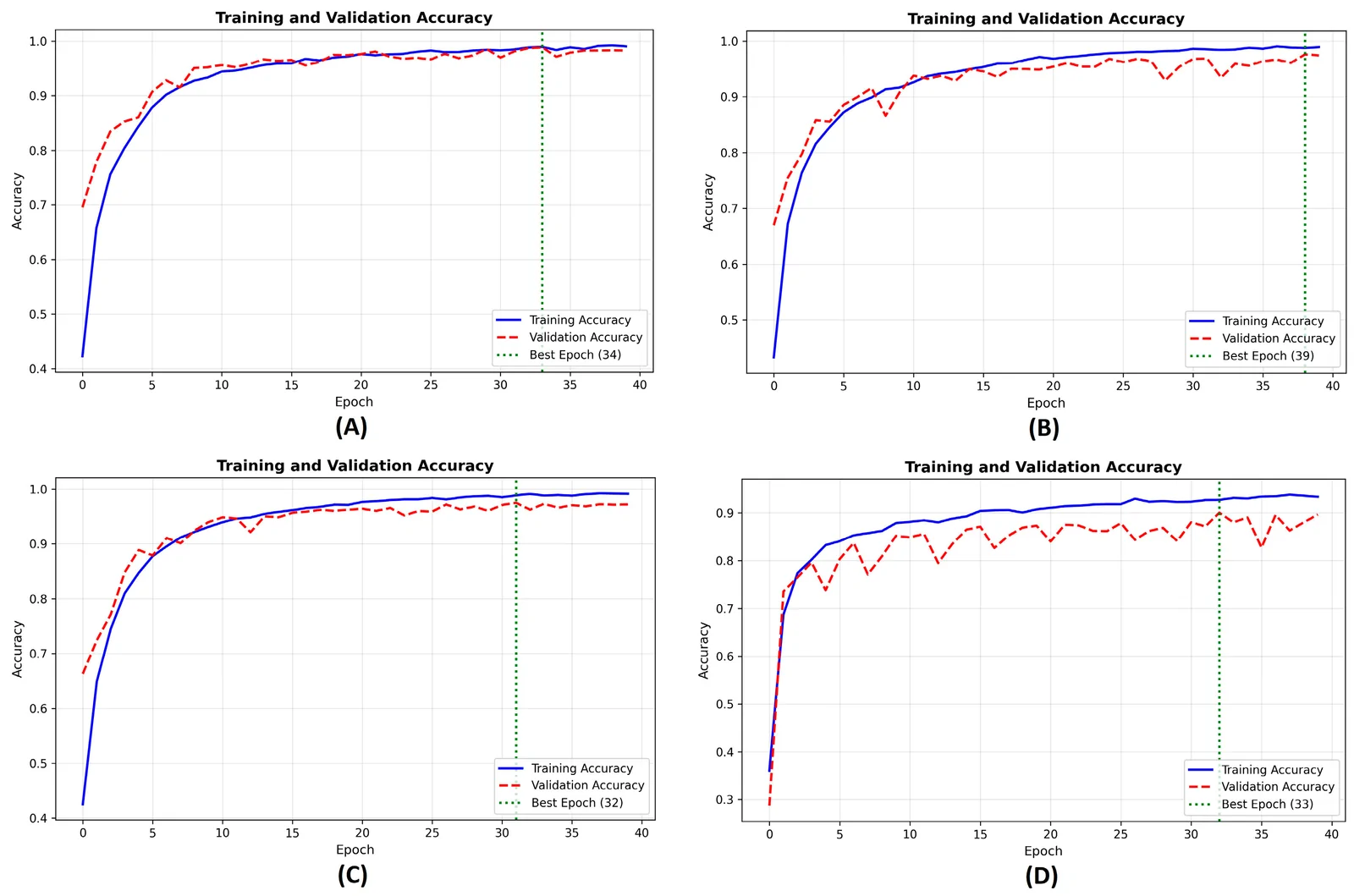
Training and validation accuracy curves are presented for (**A**) XHIC-Net, (**B**) Based-CNN, (**C**) CNN-INN, and (**D**) INN.

Training and test accuracy (across epochs) of XHIC-Net, INN, Based-CNN, and CNN-INN are compared in Figure 8. HIC-Net achieves fast convergence of test accuracy towards the maximal values with a small gap between training and test curves in both datasets. This result shows that there was better generalization and little overfitting. By contrast, VGG-16 converges more slowly and exhibits a greater discrepancy between training and test accuracies, suggesting overfitting. The INN baseline achieves an intermediate level of performance, highlighting the usefulness of the hybrid architecture constructed in XHIC-Net.

**Figure 9:**
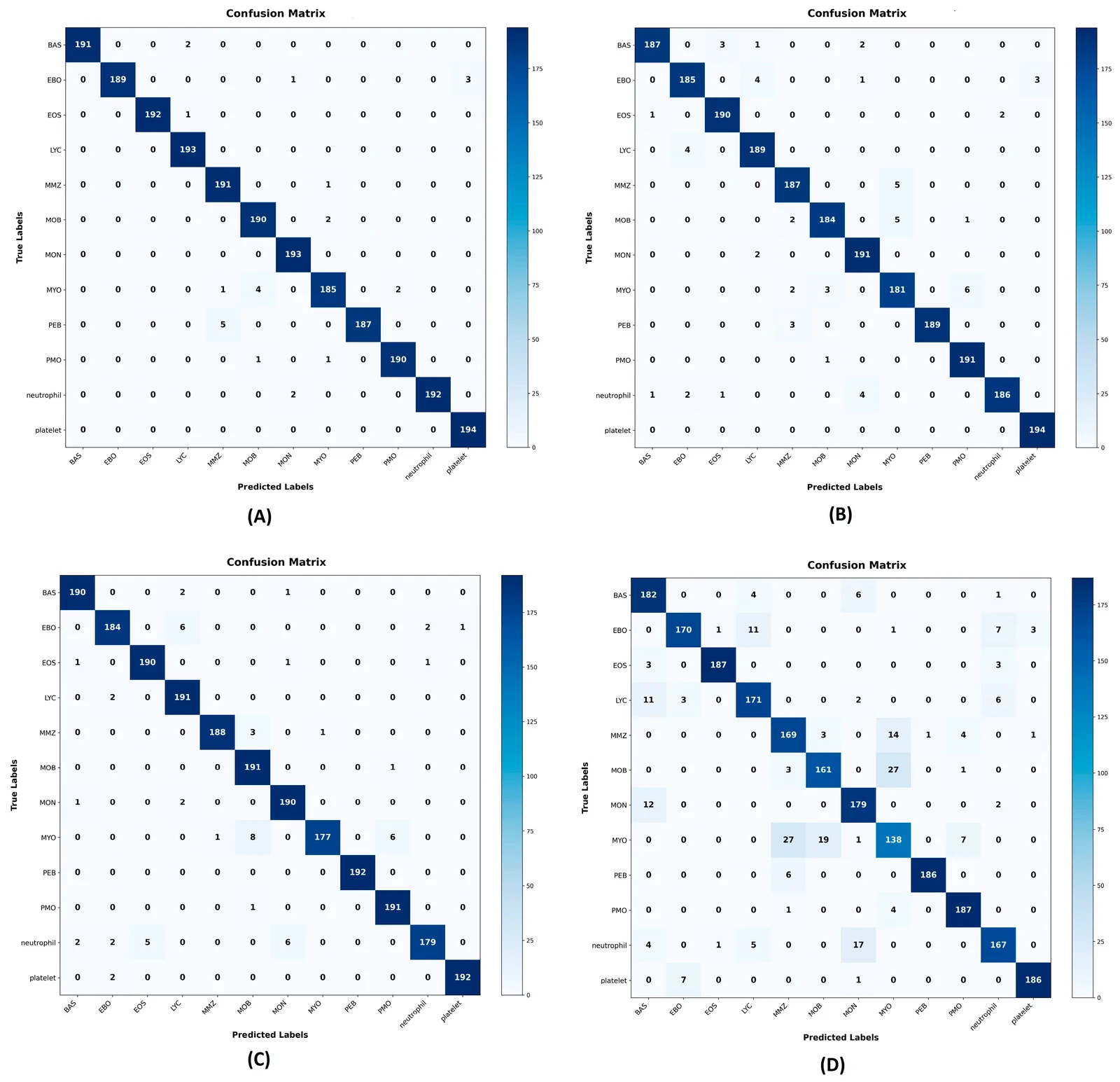
Confusion matrices corresponding to (**A**) XHIC-Net, (**B**) Based-CNN, (**C**) CNN-INN, and (**D**) INN on the 12-class combined dataset.

The confusion matrices for the evaluated models on the 12-class combined dataset are shown in Figure 9. In those matrices, the associated classes represent actual (rows) and predicted (columns), where the numbers show the count/percentage given to that particular class. The dark diagonal elements show that most classes have very high rates of correct classification and few misclassifications. The off-diagonal elements represent errors and are much smaller than the diagonal elements, especially for morphologically unrelated classes. Occasionally, visually similar classes are misclassified, indicating a challenge in separating subtle morphological features within subsamples of blood smear images. Overall, however, this visualization confirms that the models perform quite adequately in terms of multiclass discrimination, as evidenced by the clear diagonal dominance of cell types. This also signifies an increased ability to distinguish closely related cell types.

**Figure 10:**
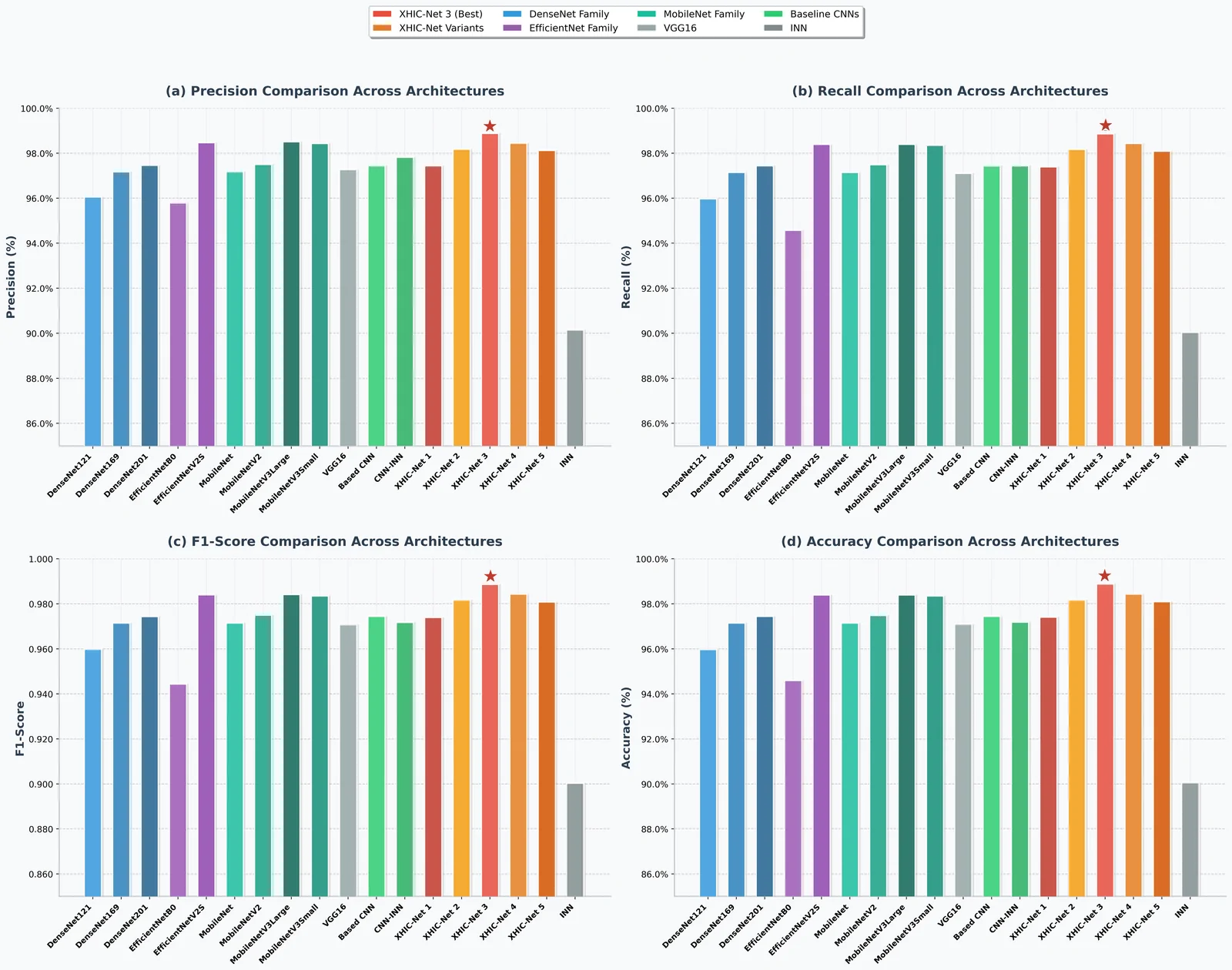
Performance comparison of the proposed XHIC-Net variants and other deep learning architectures in terms of (**a**) precision, (**b**) recall, (**c**) F1-score, and (**d**) accuracy. The red star (★) denotes XHIC-Net 3, which achieves the best performance among the evaluated architectures.

Figure 10 shows the precision, recall, accuracy, and F1 score curves, indicating the trade-off between precision and recall for different threshold settings. The results show that XHIC-Net achieves an optimal balance between high-accuracy identification and false-positive reduction, confirming its effectiveness in multiclass classification tasks.

**Figure 11:**
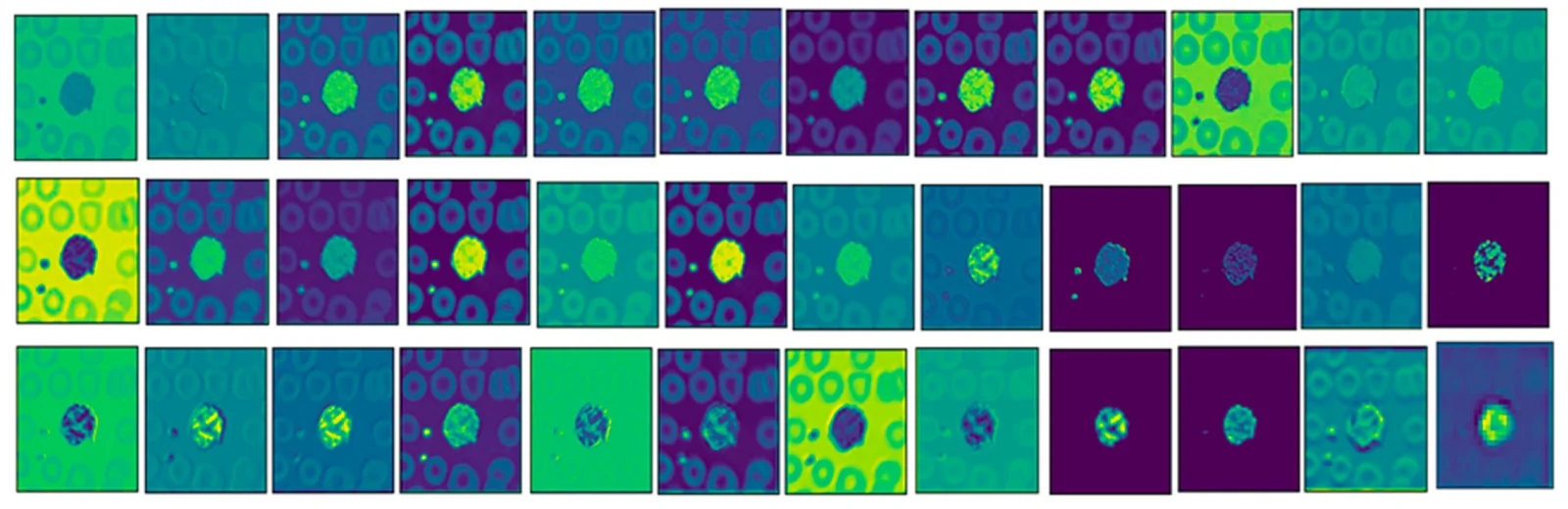
Visualization of feature maps extracted from CNN layers.

**Figure 12:**
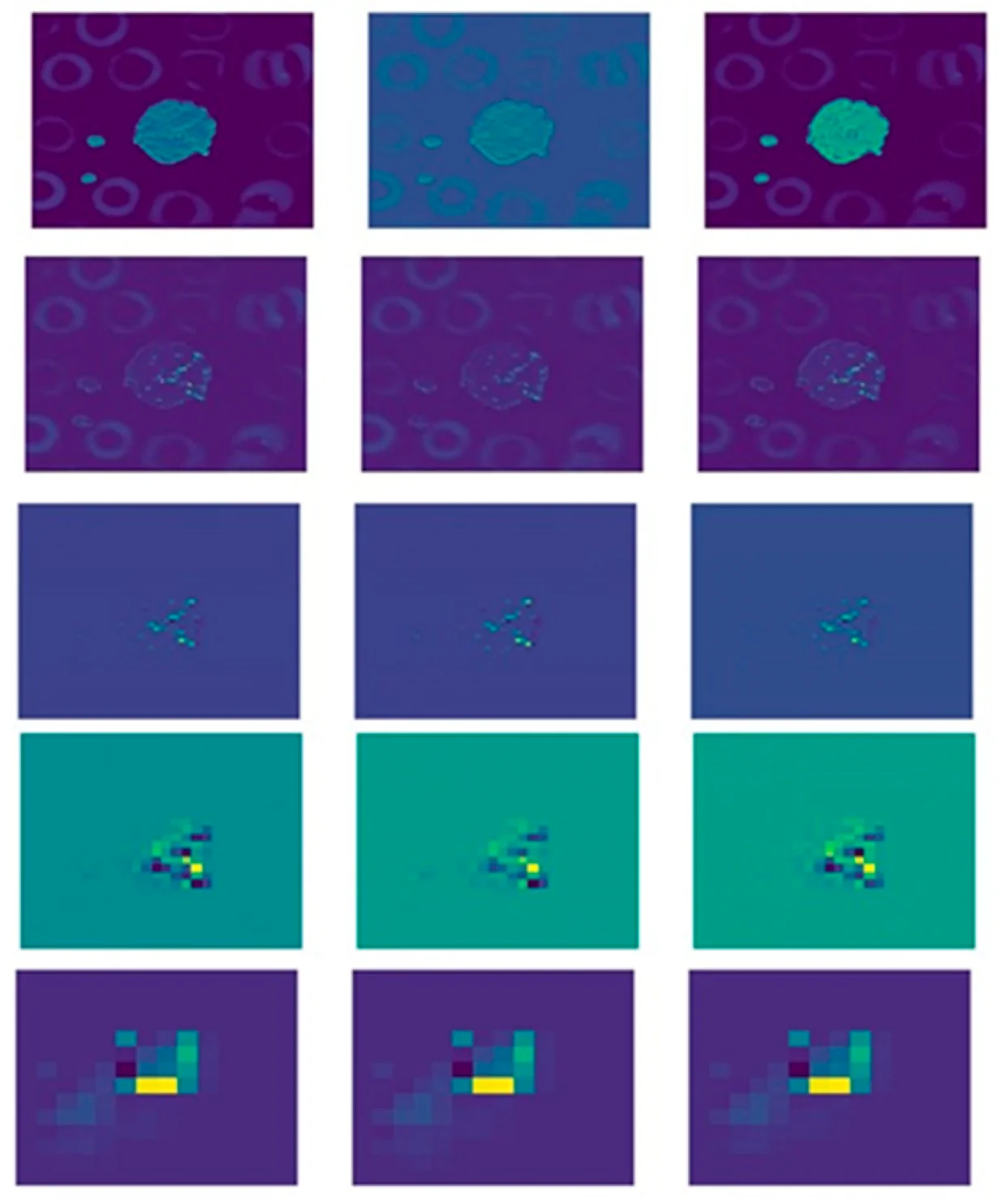
Visualization of feature maps from the layers of the INN.

Figure 11 shows different CNN visualization layers, while Figure 12 shows the feature map of the invertible INN layers implemented in the proposed model. The higher-level layers demonstrate the model’s ability to extract and reconstruct unique features from the input data. These layers reveal which specific features are highlighted and provide insight into how the model learns spatial patterns and which features are important. Together, the visualizations provide valuable insights into how well the model encodes information at different levels.

### 4.4. Explainable AI with XHIC-Net

**Figure 13:**
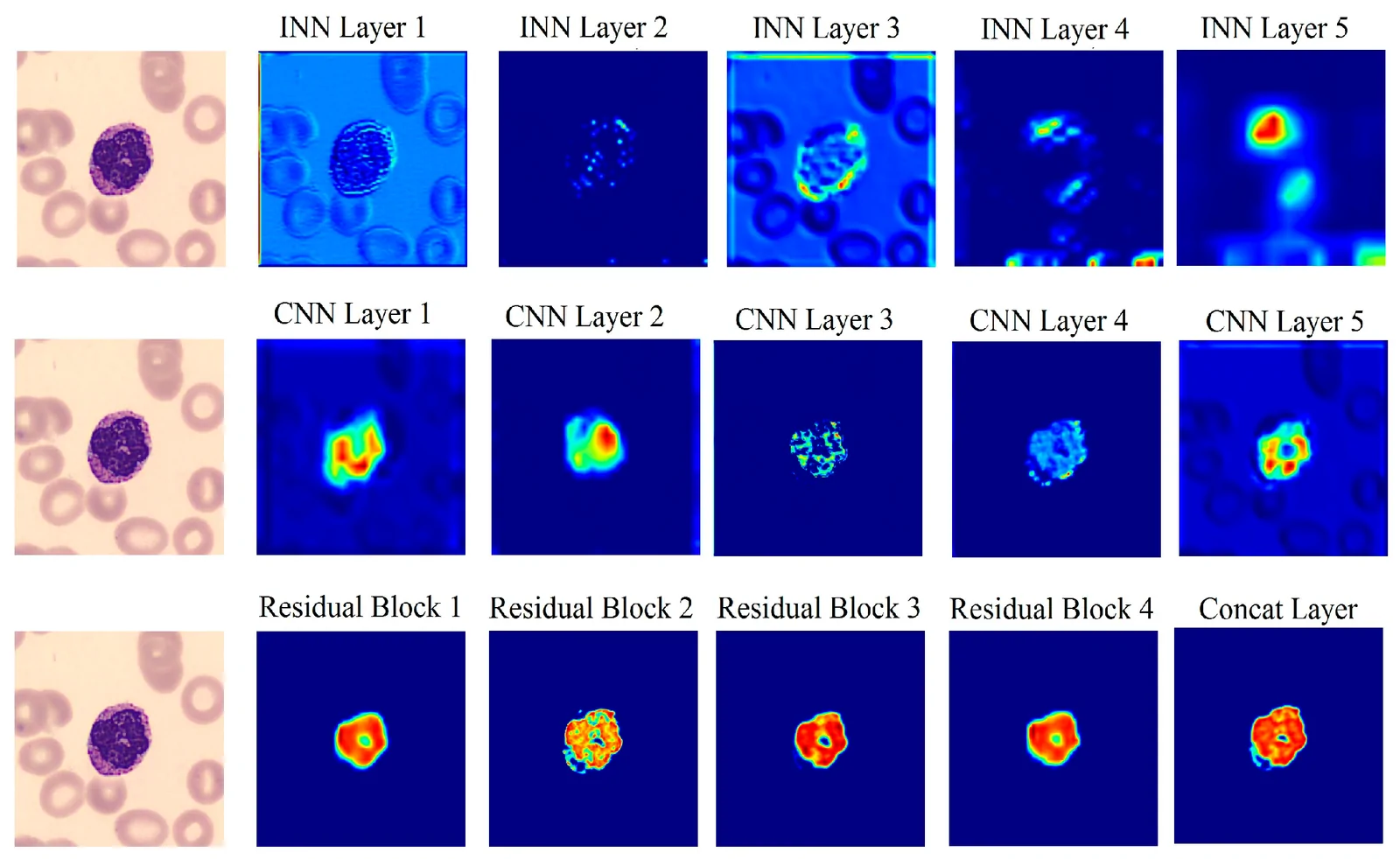
Grad-CAM visualizations illustrating explainable artificial intelligence within the XHIC-Net framework.

Finally, our hybrid, architecture-based model for classifying leukocytes and non-leukocytes in blood smears consists of involution and CNN layers, with residual blocks in between and a concatenation layer at the end. This is illustrated by the feature map visualizations in Figure 13. Involution layers 1–5 refine the spatially adaptive features learned at the previous layer, resulting in a condensed representation of the raw leukocyte images. For the fifth layer, activations form concentrated hotspots that emphasize important local information in the slider images. Meanwhile, core layers 1–5 and residual blocks 1–4 retrieve differentiating features while preserving the key characteristics and gradient flow of leukocytes through downsampling. The concatenation layer combines these complementary features to provide accurate recognition of the twelve most commonly observed leukocyte and non-leukocyte cell types in blood smear images.

We use Grad-CAM to link model predictions to biologically relevant factors. This interpretability is crucial for building trust among clinicians and enables those working in hematology to understand the rationale behind diagnosing diseases such as leukemia. The model is designed to work with input images of 224 × 224 × 3 pixels, reducing the spatial dimension to 7 × 7 whilst increasing the depth of features. It also integrates a dense layer of 256 units interspersed with dropout to enhance robustness. This research shows promise for use in computer-aided inspection of leukocytes after performing normalization on a test dataset with a given batch size as it bridges the gap between high-level neural architectures and medical diagnostics. This model is an accessible hematology tool with visualizations that help to illuminate the contribution of each layer to the final prediction.

**Figure 14:**
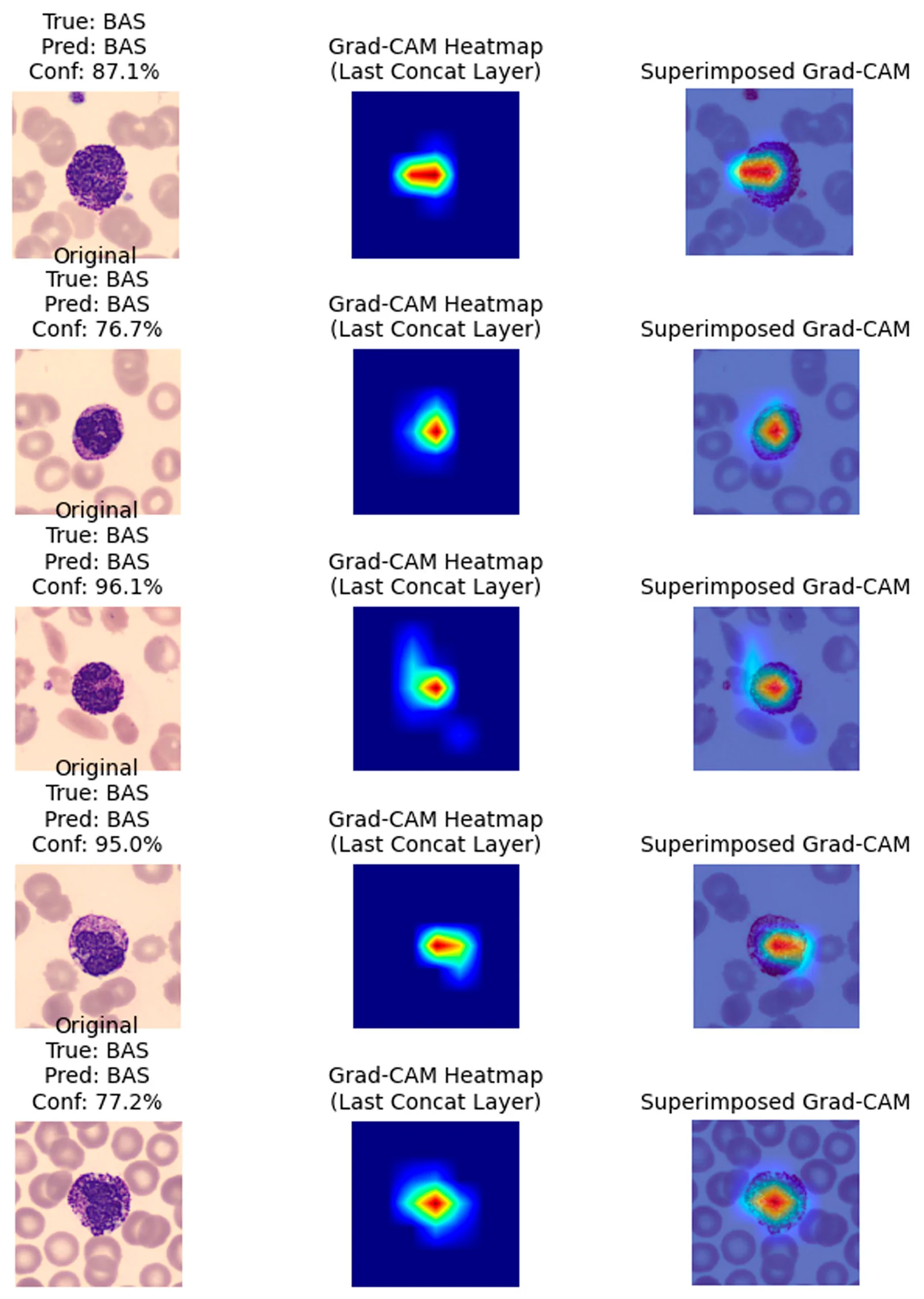
Grad-CAM visualizations, based on predictions, for basophil class samples. For each row: (Original image with true/predicted label and confidence, Grad-CAM heatmap from the last concatenate layer, superimposed Grad-CAM on original image). The recurrent feature identified by the heatmaps is nuclear shape and granule distribution—key morphological traits that hematologists rely on for cell identification.

Prediction-based Grad-CAM visualizations have been added to Figure 14. This figure displays original blood smear images, Grad-CAM heatmaps from the final concatenation layer, and superimposed heatmaps for various samples of basophils with varying confidence scores. The heatmaps clearly demonstrate the nuclear area with the surrounding cytoplasmic granules, which are the main morphological characteristics hematologists look for when identifying basophils. This was confirmed quantitatively by expert evaluation and overlap analysis.

The new visualizations show that the model concentrates on clinically relevant regions: Note intense activation surrounding the dense, lobulated nucleus and characteristic coarse granules of basophils. The model still attends to the nuclear boundary and granule distribution correctly even in scenarios with less confidence. These visualizations qualitatively link the model’s decision-making process to established hematological morphology criteria. However, quantitative clinical validation is discussed in Section 5.

### 4.5. Cross-Dataset Validation

To rigorously evaluate the generalizability of XHIC-Net across different institutions, staining protocols (Wright–Giemsa vs. May–Grünwald–Giemsa), and sample types (peripheral blood vs. bone marrow), we performed both dataset-wise evaluation and cross-dataset (leave-one-dataset-out) validation.

The main model trained on the combined 12-class dataset achieved excellent performance when evaluated separately on each source: 98.23% accuracy on the seven overlapping classes from Acevedo et al. [47] and 98.23% accuracy on the five classes from Matek et al. [48]. Additionally, we conducted leave-one-dataset-out validation by training the model exclusively on one dataset and testing on the other using the overlapping classes. These experiments (Table 10) demonstrate that XHIC-Net maintains high performance across both data sources, indicating that the model primarily learns robust cell morphological features rather than dataset-specific artifacts.

**Figure 15:**
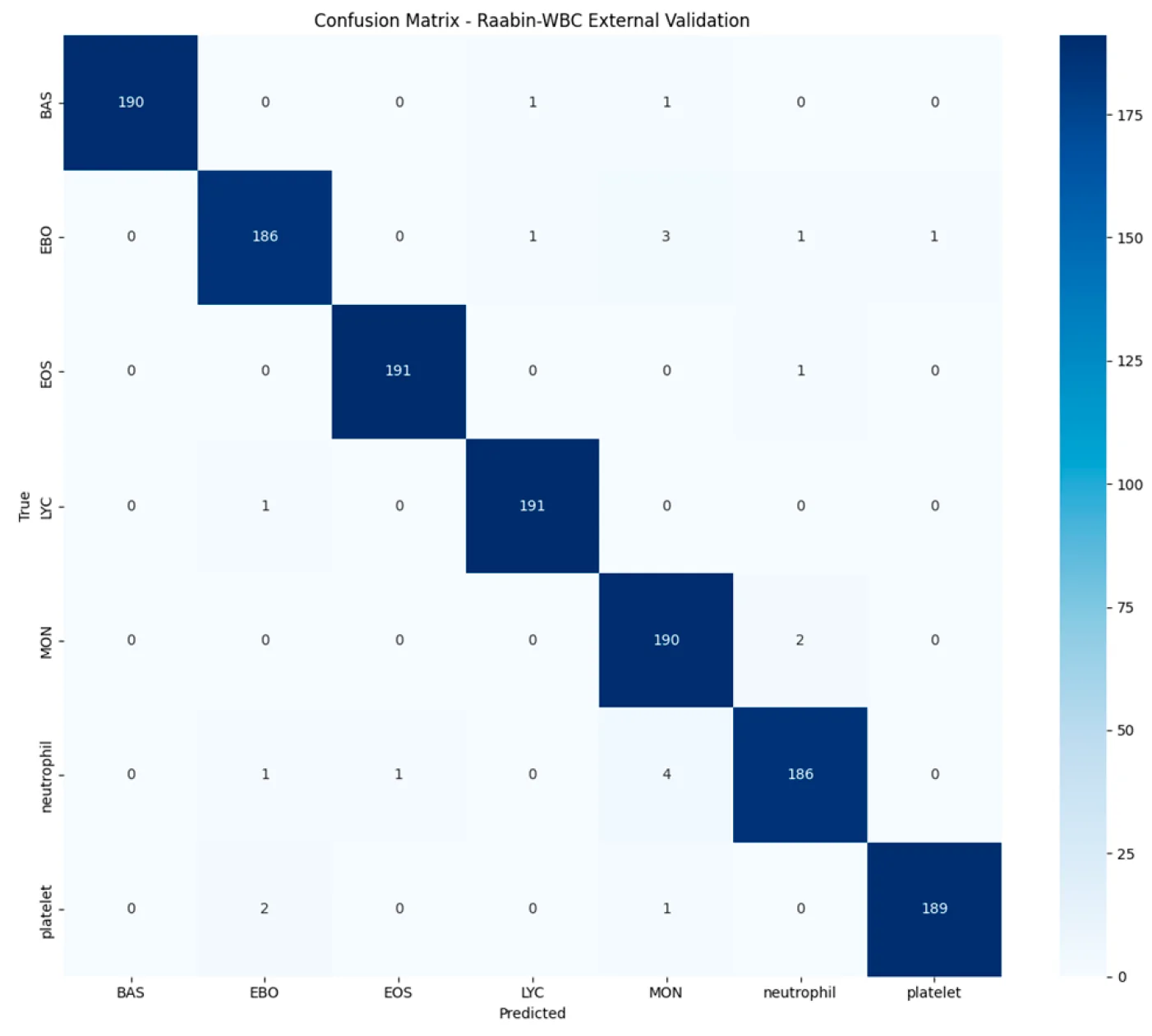
Confusion matrix results using cross-validation.

In Table 10, we show that the XHIC-Net preserves excellent performance on the Acevedo external dataset, reaching an accuracy of 98.44%. This small drop from the original results is not unexpected, as there are likely some differences in imaging protocols, staining, or patient composition between the datasets. The robust and consistent performance of the hybrid involution–convolution architecture across different datasets validates its generalizability to various data sources and clinical applications. Figure 15 shows the confusion matrix results for the Acevedo et al. dataset. These were obtained using cross-validation and the same model weights.

### 4.6. Ablation Study

The receptive field configuration of the involution operation was systematically explored to determine the best spatial size for feature aggregation, as shown in Table 8. The 7 × 7 kernel configuration was found to be the best option, with an accuracy of 98.36%, an error rate of 1.64%, and an inference latency of 5.49 ms. The 5 × 5 kernel performed poorly, achieving an accuracy of just 97.62%, due to its inability to capture the morphology of the lesions through contextual aggregation. By contrast, the 9 × 9 kernel achieved an accuracy of 98.18%. This slight decrease is probably due to excessive spatial smoothing, which reduces fine-grained pathological signatures. These results justify the choice of 7 × 7 as the default kernel size.

We performed a reduction-ratio ablation to validate the channel compression in XICH-Net, as shown in Table 9. Ratio 3 achieved a test accuracy of 98.14% vs. 97.92% for ratio 1, with computational overhead nearly equaling (64.88 s vs. 68.98 s training time per epoch). This improvement suggests that the proposed block uses a moderate amount of channel reduction to enhance representational efficiency by suppressing redundant activations while preserving features that are important for diagnosis.

## 5. Discussion

The proposed XHIC-Net model, which employs a hybrid involution–convolution network-based residual block architecture, demonstrates superior performance in multiclass classification of morphological cell types of leukemia. XHIC-Net achieves 98.88% accuracy, with precision 98.89%, recall 98.87%, and Cohen’s Kappa score of 0.9877, on a comprehensive dataset containing image specimens from various leukocyte subtypes acquired via several microscopic imaging techniques. These results are better than those of all the other methods and evaluated algorithms. With the same level of performance as other DL models for 12 classes, XHIC-Net is more efficient with fewer parameters and is more scalable. XHIC-Net is an efficient choice for deployment in settings where resources are limited, thanks to its combination of reduced parameters and the significant speed boost provided by its lightweight design.

This dataset-wise analysis shows that XHIC-Net performance is due to learned cell morphology rather than dataset-specific artifacts. The low performance difference between Wright–Giemsa and May–Grünwald–Giemsa-stained images indicates staining insensitivity, making clinical application feasible in laboratories using diverse protocols.

Several limitations are present in these studies. Although the dataset is comprehensive, it may be biased due to its derivation from a single healthcare institution (Barcelona, 2015–2019) and the Munich Leukemia Laboratory. As a result, the generalizability of the findings to other populations and imaging environments is limited. The model has not been thoroughly evaluated on blurred or low-quality images, which could affect its robustness in clinical settings with variable image quality. Furthermore, the computational advantages observed may not persist when applied to significantly larger datasets or more complex imaging scenarios, necessitating further validation.

In regular clinical practice, disagreements can arise among hematologists regarding borderline or immature cells due to varied morphology, staining artifacts, and the overlap in morphological indications between reactive and leukemic cells. The model could miss false negatives, especially in early disease or when immature blasts are morphologically similar to normal or reactive cells. We recommend prospective multi-center validation against multiple hematologists’ diagnoses on independent datasets in future clinical studies to address this limitation. Moreover, a thorough error analysis with an emphasis on clinically significant classifications—most notably, false negatives in leukemia predictions—will be crucial for enhancing model robustness and reducing the risk of missing out on critical cases. Such analyses will help to define confidence intervals for real-world performance and guide the development of effective human–AI hybrid diagnostic systems.

XHIC-Net shows great potential for clinical application. The model is highly accurate and efficient in predicting the presence of hematological diseases, particularly leukemia, and can automatically classify leukocytes from peripheral blood smears. This approach reduces the time and expertise required for detailed analysis. This could potentially reduce medical expenses. It could also improve personalized healthcare. This is particularly true in settings with limited resources. The model’s adaptability to other medical imaging tasks, including cancer cell detection and tissue analysis, merits further investigation through the incorporation of adaptive kernel sizes and enhanced normalization techniques, as outlined in future research proposals. Table 11 compares the SOTA with the proposed work.

Limitations of XAI validation. Although the Grad-CAM visualizations in Figure 13 and Figure 14 are qualitatively indicative of biological interpretability, we caution that these claims do not have quantitative validation against expert annotation. In this study, saliency maps and grading were evaluated by three independent hematopathologists using standardized scoring. However, quantitative inter-rater agreement metrics were not provided, nor was there a comparison against manual segmentation of nuclei and granules. Future work will inevitably focus on this area to objectively demonstrate agreement between model attention and established morphological criteria, thereby enhancing clinical trust in automated predictions.

XHIC-Net shows good performance on retrospective public datasets, but there are some obstacles to clinical translation. First, the study is based solely on publicly available datasets [47,48] and not on prospective data from clinical sites. Second, there was no external prospective validation of newly acquired patient samples. Thirdly, the evaluation was performed using single-cell cropped images rather than whole-slide blood smear images. This avoided the issues associated with cell detection, segmentation, and identification of rare cells within the clinical workflow. Fourth, the deployment aspects like inference latency on edge devices, integration with laboratory information systems, and regulatory approval pathways were not discussed. These restrictions make XHIC-Net a research prototype that has clinical potential, but not a deployable diagnostic device.

## 6. Conclusions

The proposed hybrid model demonstrates high suitability for classifying leukocyte and non-leukocyte blood smears, having been trained on a dataset containing 12 classes and 12,879 images. The model achieved an overall accuracy and precision of 98.88%, along with excellent recall and a high Cohen’s Kappa score, thereby surpassing most existing methods. It also outperformed several dense-layered pretrained models, including MobileNetV3Large 98.40%, MobileNetV3Small 98.36%, and EfficientNetV2S 98.40%. The lightweight architecture enables faster processing, supports real-time clinical applications, and reduces computational costs. Nevertheless, potential dataset bias and limited evaluation on low-quality images remain areas requiring further investigation. In summary, XHIC-Net constitutes a substantial advancement in automated medical image analysis, with significant potential to enhance patient care and diagnostic efficiency. XHIC-Net not only outperforms existing models in accuracy but also demonstrates superior computational efficiency with lower inference time and memory footprint.

In future work, we plan to optimize the hyperparameters of our XHIC-Net model, including kernel sizes and reduction ratios for the two attention modules, using genetic algorithms. This method is expected to enhance the accuracy and computational efficiency of analyzing heterogeneous leukocyte samples with complex morphologies. Using genetic algorithms for hyperparameter optimization should further improve model performance and robustness by enabling more comprehensive learning of morphological diversity, such as variations in monocyte size and abnormal blast forms, which are frequently observed in heterogeneous leukocytes. The use of transformers is also supported by their demonstrated effectiveness in capturing long-range dependencies in medical imaging, thereby increasing the model’s ability to learn complex interclass relationships within large leukocyte datasets. Collectively, these advancements are expected to improve the stability of our model and extend its applicability to a broader range of diagnostic imaging tasks.

## Figures and Tables

**Table 1:** Original class distribution in the Matek et al. dataset before selection and curation.

Class	Images	Class	Images
Band neutrophils	9968	Artifacts	27,395
Segmented neutrophils	29,424	Not identifiable	409
Lymphocytes	26,242	Erythroblasts	8
Monocytes	4040	Hairy cells	65
Eosinophils	5883	Abnormal eosinophils	47
Basophils	11,994	Immature lymphocytes	2740
Metamyelocytes	11,973	Faggot cells	6557
Promyelocytes	7629	Proerythroblasts	27,395
Blasts	42	Myelocytes	737
Plasma cells	294	Other cells	11,973
Smudge cells	11,994		

**Table 2:** Distribution of Classes in the Combined Dataset.

Class	Validation	Test	Train
BAS	111	193	801
EBO	110	193	793
EOS	112	193	818
LYC	107	193	767
MMZ	104	192	739
MOB	103	192	733
MON	107	193	774
MYO	103	192	729
PEB	103	192	737
PMO	105	192	751
neutrophil	109	194	783
platelet	116	194	851
**Total**	**1290**	**2313**	**9276**

**Table 3:** Summary of ablation studies on baseline CNN and INN vs. the proposed hybrid XHIC-Net architecture. The performance metrics, number of parameters, and training heatmap are provided to assess the individual contribution of each modular component.

Block/Stage	Main Operation	Purpose	Parameters
Input	224 × 224 × 3 RGB image	Raw microscopic blood smear image input	0
Convolutional Branch	5× Conv2D + 3 Residual Blocks + ReLU	Extracts hierarchical features using standard convolution	106,570
Involution Branch	5 Involution layers + ReLU + MaxPool	Captures spatially adaptive and channel-specific features	130
Feature Fusion	Concatenation of both branches	Combines complementary features from convolution and involution	0
Classification Head	Flatten + 2 Dense layers + Dropout + Softmax	Produces probability scores for 12 cell classes	307,468

**Table 4:** Algorithm for the Involution Neural Network (INN) in TensorFlow.

**TensorFlow Involution Layer**
**1. Input Parameter:** $C:\dot{channel}$*s*, G:group*s*,
K:kernel size,δ:stride,r:reduction ratio
**2. Build phases:**
$compute\;H=H_{in⁄s}$ $,\;w=w_{in⁄s}$
Stride layer
$o=AvgPool2D\left(s,s\right)if\;s>1\;else\;Identity$
Kernel generation:
Sequential layer
Conv2DCr,1→BatchNorm→ReLu→Con2DK.K.G.I.
Reshape layer
$reshape\;kernel\;to\;[H,W,K^{2},1,G]$.
$reshape\;patches\;to\;[H,W,K^{2},C⁄C,G]$.
reshape output to [H,W,C].
**3. Forward pass:**
Kernel generation
kernel=Reshape(kernelGen(o(x,K,s))).
Input patches
Extract patches=ExtractPatches(x,K,s)
$Reshapepatchesto\left[H,W,K^{2},C/G,G\right].$
Multiply to add
$Output=sum\left(kernel.patches,axis=3\right).$
Reshape output to[H,W,C]

**Table 6:** Computational complexity comparison of XHIC-Net with baseline models.

Method	No of Param	Weight MB	Average Time/Epoch	Inference Time (ms/Image)	FPS	GPU Memory (MB)
DenseNet121	7,049,804	28.1	40.80 s	6.69	149.4	6913
DenseNet169	12,662,860	50.0	48.99 s	7.75	129.0	6877
DenseNet201	18,345,036	72.0	73.39 s	10.25	97.6	7107
EfficientNetB0	4,064,943	15.9	95.71 s	7.00	142.9	7317
EfficientNetV2S	20,346,732	234	295.45 s	8.54	117.1	7271
MobileNet	3,241,164	12.7	26.56 s	5.17	193.6	6957
MobileNetV2	2,273,356	9.25	26.79 s	5.17	193.3	6969
MobileNetV3Large	3,007,884	35.4	55.43 s	6.10	163.9	7408
MobileNetV3Small	946,044	11.7	43.51 s	5.68	176.0	7377
VGG16	14,720,844	56.2	152.75 s	7.54	132.6	7400
Based CNN	296,172	3.46	26.14 s	4.87	205.4	7422
CNN-INN	334,104	1.38	33.11 s	5.46	183.1	7390
XHIC-Net 1	360,792	1.49	58.92 s	5.59	178.8	7157
XHIC-Net 2	387,480	1.71	63.56 s	5.43	184.1	7254
XHIC-Net 3	414,168	1.96	66.26 s	5.56	180.0	7250
XHIC-Net 4	420,856	2.05	66.37 s	5.61	178.2	7184
XHIC-Net 5	427,544	2.19	66.42 s	5.55	180.2	7230
INN	107,064	0.50	32.04 s	5.33	187.8	7507

**Table 7:** Pairwise McNemar’s comparison with effect sizes.

Comparison	McNemar’s χ^2^	*p*-Value	Cohen’s h	95% CI (h)
XHIC-Net 3 vs. CNN-INN	37.03	<0.001	0.125	0.104–0.145
XHIC-Net 3 vs. CNN	31.03	<0.001	0.109	0.088–0.129
XHIC-Net 3 vs. XHIC-Net 1	32.03	<0.001	0.111	0.091–0.132
XHIC-Net 3 vs. XHIC-Net 2	13.07	<0.001	0.056	0.035–0.076
XHIC-Net 3 vs. XHIC-Net 4	8.10	0.00443	0.038	0.018–0.059
XHIC-Net 3 vs. XHIC-Net 5	16.06	<0.001	0.065	0.044–0.085
XHIC-Net 3 vs. INN	202.00	<0.001	0.429	0.409–0.450
XHIC-Net 3 vs. DenseNet121	65.01	<0.001	0.192	0.171–0.212
XHIC-Net 3 vs. DenseNet169	38.02	<0.001	0.127	0.107–0.148
XHIC-Net 3 vs. DenseNet201	31.03	<0.001	0.109	0.088–0.129
XHIC-Net 3 vs. EfficientNetB0	97.01	<0.001	0.257	0.237–0.277
XHIC-Net 3 vs. EfficientNetV2S	9.09	0.00257	0.042	0.021–0.062
XHIC-Net 3 vs. MobileNet	38.02	<0.001	0.127	0.107–0.148
XHIC-Net 3 vs. MobileNetV2	30.03	<0.001	0.106	0.086–0.127
XHIC-Net 3 vs. MobileNetV3Large	9.09	0.00257	0.042	0.021–0.062
XHIC-Net 3 vs. MobileNetV3Small	10.08	0.00150	0.045	0.024–0.065
XHIC-Net 3 vs. VGG16	39.02	<0.001	0.130	0.110–0.151

**Table 8:** Ablation on involution kernel size.

Kernel Size	Training Time/Epoch	Inference Time	Accuracy	Error Rate	Precision	Recall	F1-Score	Cohen’s Kappa
5 × 5	68.51 s	6.13 ms	97.62%	2.38%	97.63%	97.62%	0.9761	0.9741
7 × 7	69.02 s	5.49 ms	98.36%	1.64%	98.37%	98.36%	0.9835	0.9821
9 × 9	64.51 s	5.37 ms	98.18%	1.82%	98.20%	98.18%	0.9819	0.9802

**Table 9:** Ablation on the reduction ratio.

Reduction Ratio	Training Time/Epoch	Inference Time	Accuracy	Error Rate	Precision	Recall	F1-Score	Cohen’s Kappa
1	68.98 s	5.75 ms	97.92%	2.08%	97.94%	97.92%	0.9791	0.9774
3	64.88 s	5.37 ms	98.14%	1.86%	98.15%	98.14%	0.9814	0.9797

**Table 10:** Performance comparison of the main XHIC-Net model on the combined 12-class dataset versus dataset-wise and cross-dataset evaluations. All results confirm strong generalization across the two public data sources with different staining and acquisition conditions.

Metric	Original 12-Class Dataset	Cross-Dataset Validation (7 Classes)	Test Results on 7 Classes [47]	Test Results on 5 Classes [48]
Accuracy (%)	98.88	98.44	98.23%	98.23%
Precision (%)	98.89	98.45	98.25%	98.23%
Recall (%)	98.87	98.44	98.23%	98.23%
Cohen’s Kappa	0.9877	0.9818	0.9779	0.9793

**Table 11:** Comparison between XHIC-Net and state-of-the-art (SOTA) methods.

Study	Methods	Classes	Accuracy
Ghosh et al. [24]	DenseNet121, ResNet50, & LEC	4	96.67%
Emrah ASLAN et al. [29]	ResNet-50, VGG19, QDA	4	98.27%
Yingying Ding et al. [21]	ResNet + SVM	10	97.03%
Channabasava Chola et al. [30]	BCNet	8	98.51%
Banik et al. [38]	CNN Model	4	96%
C. Zonyfar et al. [39]	CAB-Net	8	98.29%
Proposed Work	XHIC-Net	12	98.88%

## Data Availability

1. https://www.kaggle.com/datasets/unclesamulus/blood-cells-image-dataset (accessd on 20 July 2025) 2. https://www.cancerimagingarchive.net/collection/bone-marrow-cytomorphology_mll_helmholtz_fraunhofer/ (accessd on 21 July 2025) (OR) https://www.kaggle.com/datasets/andrewmvd/bone-marrow-cell-classification?select=bone_marrow_cell_dataset (accesson on 25 July 2025)

## Abbreviations

The following abbreviations are used in this manuscript:

XHIC-Net	Explainable Hybrid Involution–Convolution Network
ACS	American Cancer Society
ML	Machine Learning
ANNs	Artificial Neural Networks
DL	Deep Learning
CNN	Convolutional Neural Network
WBCs	White Blood Cells
BAS	Basophil
EBO	Erythroblast
EOS	Eosinophil
LYC	Lymphocyte
MMZ	Metamyelocyte
MOB	Monoblast
MON	Monocyte
MYO	Myeloblast
PEB	Proerythroblast
PMO	Promyelocyte
INN	The Involution Neural Network
TP	True Positive
TN	True Negative
FP	False Positive
FN	False Negative
MLL	Munich Leukemia Laboratory
DC-GAN	Deep Convolutional Generative Adversarial Network
CI	Confidence Intervals
CDNN	Custom Deep Neural Network
